# Ucp1 Ablation Improves Skeletal Muscle Glycolytic Function in Aging Mice

**DOI:** 10.1002/advs.202411015

**Published:** 2024-11-21

**Authors:** Jin Qiu, Yuhan Guo, Xiaozhen Guo, Ziqi Liu, Zixuan Li, Jun Zhang, Yutang Cao, Jiaqi Li, Shuwu Yu, Sainan Xu, Juntong Chen, Dongmei Wang, Jian Yu, Mingwei Guo, Wenhao Zhou, Sainan Wang, Yiwen Wang, Xinran Ma, Cen Xie, Lingyan Xu

**Affiliations:** ^1^ Shanghai Key Laboratory of Regulatory Biology Institute of Biomedical Sciences and School of Life Sciences East China Normal University Shanghai 200241 China; ^2^ State Key Laboratory of Drug Research Shanghai Institute of Materia Medica Chinese Academy of Sciences Shanghai 201203 China; ^3^ School of Chinese Materia Medica Nanjing University of Chinese Medicine Nanjing 210023 China; ^4^ University of Chinese Academy of Sciences Beijing 100049 China; ^5^ Department of Endocrinology and Metabolism Fengxian Central Hospital Affiliated to Southern Medical University Shanghai 201499 China; ^6^ Shanghai Frontiers Science Center of Genome Editing and Cell Therapy Shanghai Key Laboratory of Regulatory Biology and School of Life Sciences East China Normal University Shanghai 200241 China; ^7^ Chongqing Key Laboratory of Precision Optics Chongqing Institute of East China Normal University Chongqing 401120 China

**Keywords:** aging, creatine, glycolytic function, skeletal muscle, thermogenic adipose tissue

## Abstract

Muscular atrophy is among the systematic decline in organ functions in aging, while defective thermogenic fat functionality precedes these anomalies. The potential crosstalk between adipose tissue and muscle during aging is poorly understood. In this study, it is showed that UCP1 knockout (KO) mice characterized deteriorated brown adipose tissue (BAT) function in aging, yet their glucose homeostasis is sustained and energy expenditure is increased, possibly compensated by improved inguinal adipose tissue (iWAT) and muscle functionality compared to age‐matched WT mice. To understand the potential crosstalk, RNA‐seq and metabolomic analysis were performed on adipose tissue and muscle in aging mice and revealed that creatine levels are increased both in iWAT and muscle of UCP1 KO mice. Interestingly, molecular analysis and metabolite tracing revealed that creatine biosynthesis is increased in iWAT while creatine uptake is increased in muscle in UCP1 KO mice, suggesting creatine transportation from iWAT to muscle. Importantly, creatine analog β‐GPA abolished the differences in muscle functions between aging WT and UCP1 KO mice, while UCP1 inhibitor α‐CD improved muscle glycolytic function and glucose metabolism in aging mice. Overall, these results suggested that iWAT and skeletal muscle compensate for declined BAT function during aging via creatine metabolism to sustain metabolic homeostasis.

## Graphical abstract

In aging UCP1^‐/‐^ mice, brown fat function declines, while beige fat‐specific creatine biosynthesis is increased to sustain its mitochondrial function. Besides, the enhanced secreted creatine from beige fat is taken up by skeletal muscle and compensatorily improves muscle glycolytic function, thereby increasing energy metabolism.

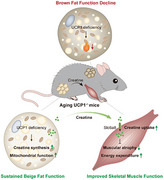

## 1 Introduction

Adipose tissues, including white, brown and beige fat, play crucial roles in regulating glucose homeostasis and energy metabolism.^[^ ^1^, ^2^ ^]^ Brown and beige fats are thermogenic fats that drive heat production and energy expenditure by consuming circulating energy substrates (i.e., glucose and lipids) through mitochondrial oxidative machinery. Uncoupling protein 1 (UCP1), an extensively studied thermogenic effector, is a key regulator of cold‐stimulated thermogenesis that functions by decoupling mitochondrial respiratory chain from intermembrane proton gradient to generate heat.^[^ ^3^, ^4^ ^]^ Besides adipose tissues, skeletal muscle is also an important tissue for both shivering and non‐shivering thermogenesis and a major contributor to glucose metabolism and energy expenditure. Numerous studies have shown the crosstalk between adipose tissue and skeletal muscle during different physiological and pathological stresses.^[^ ^5^, ^6^, ^7^, ^8^ ^]^ However, it is not clear how this crosstalk works in the aging scenario.

Aging is characterized by a systematic decline in organ functions. During aging, thermogenic fats undergo programmed loss of thermogenic capacity, especially a significant decrease in UCP1 expression and function as an early onset event of aging.^[^ ^9^, ^10^ ^]^ Notably, at late age, skeletal muscles experience sarcopenia that characterizes profound muscle mass loss and functional decline.^[^ ^11^, ^12^ ^]^ This suggests that thermogenic fat function decline precedes muscle sarcopenia. However, whether and how thermogenic fats communicate with skeletal muscle for the chronobiology of aging has not been well elucidated.

Creatine is synthesized *de novo* from three essential amino acids, arginine, glycine and methionine, or obtained from exogenous sources, such as diet or supplementation.^[^ ^13^ ^]^ Creatine is important for beige fat function since the creatine futile cycle, a creatine‐dependent substrate cycling propelled by mitochondrial ATP synthesis, is an important UCP1‐independent thermogenic mechanism that enhances energy metabolism and thermogenesis in beige fat.^[^ ^14^ ^]^ Meanwhile, as a high energy demanding tissue, the function of skeletal muscle is also tightly coupled with creatine.^[^ ^15^ ^]^ For instance, creatine increases phosphocreatine content that serves as phosphate bond reservoir to sustain ATP production, thus is critical for energy metabolism and muscle functionality.^[^ ^16^ ^]^ Besides, creatine modulates myogenesis by directly activating myogenic regulators and promoting satellite cell activation, proliferation, and differentiation.^[^ ^17^ ^]^ In addition, creatine also shows anti‐catabolic capability and promotes muscle protein synthesis via IGF1‐PI3K‐Akt‐mTOR pathway.^[^ ^18^, ^19^ ^]^ Consistent with the important role of creatine in muscle function, creatine is commonly used as a dietary supplement to enhance exercise performance in bodybuilding.

In the present study, we demonstrated that though UCP1‐deficiency (UCP1^−/−^) in aging mice caused brown fat dysfunction as expected, they maintained glucose and energy homeostasis compared to aging WT mice attributed to creatine‐mediated resistance to iWAT function decline and skeletal muscle atrophy. Of note, we identified a crosstalk between beige fat and muscle via creatine, which was blocked by creatine analog β‐guanidinopropionic acid (β‐GPA) treatment. Additionally, UCP1 inhibitor α‐CD administration led to improved muscle glycolytic function and glucose metabolism in aging mice. All these findings suggest the importance of beige fat‐skeletal muscle crosstalk for systemic energy homeostatic maintenance during aging.

## 2 Results

### 2.1 UCP1 Deficiency Impaired Lipid Metabolism While Maintained Glucose Metabolism and Energy Expenditure in Aging Mice

We first examined the expression pattern of the thermogenic marker *Ucp1* in iWAT of mice at various ages. This revealed a gradual and significant decrease in *Ucp1* levels along the advancement of aging (Figure S1A, Supporting Information), which is in consistent with previous study showing that aging leads to a programmed loss of thermogenic gene expression in murine iWAT.^[^ ^11^ ^]^ In comparison, the enhancements in muscular atrophic gene *MuRF‐1* expression in skeletal muscle only become apparent in late stage of life (Figure S1B, Supporting Information), suggesting that thermogenic fat function decline preceded muscle atrophy during aging.

**Figure 1:**
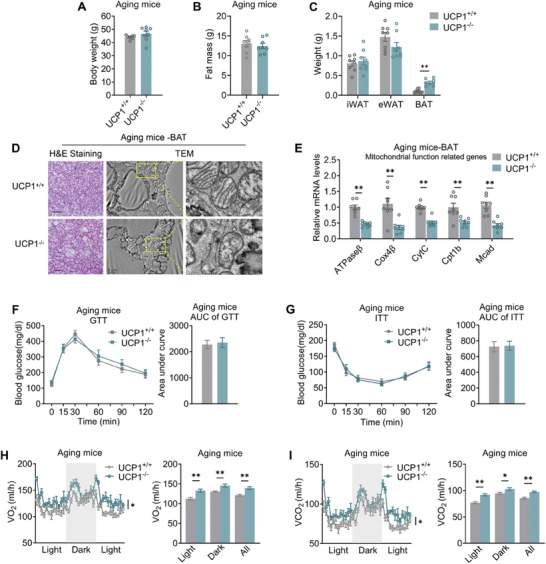
UCP1 deficiency impaired lipid metabolism while maintained glucose metabolism and energy expenditure of aging mice. A–C) Body weight A), fat mass B) and weights of adipose tissues C) of 12‐month‐old aging UCP1^+/+^ and UCP1^−/−^ mice. D) H&E staining and transmission electron microscopy (TEM) analysis of BAT from aging UCP1^+/+^ and UCP1^−/−^ mice. E) Mitochondrial gene levels of BAT from aging UCP1^+/+^ and UCP1^−/−^ mice. F,G) GTT F) and ITT G) assay and AUC quantification of aging UCP1^+/+^ and UCP1^−/−^ mice. Mice were injected intraperitoneally with glucose at 1.5 g kg^−1^ body weight for GTT and insulin at 0.75 U/kg body weight for ITT. H,I) Oxygen consumption (VO_2_, H) and Carbon dioxide excretion (VCO_2_, I) were measured using CLAMS in aging UCP1^+/+^ and UCP1^−/−^ mice. Data are presented as mean ± SEM and *p < 0.05, **p < 0.01 compared to aging UCP1^+/+^ group. *n* = 8 per group. Scale bars are 45 µm for H&E staining and 1 µm for TEM analysis.

It has been reported that UCP1^−/−^ mice are not obese, or even leaner, than the wild‐type (WT) littermates under ambient temperature in young mice.^[^ ^20^, ^21^, ^22^ ^]^ Considering that UCP1 deficiency in mice accelerated thermogenic decline, it would be important to investigate the metabolic consequences of UCP1 loss in aging mice. Detailed examination revealed that there were no significant difference in body weight, fat mass or lipid deposition in epidydimal white fat (eWAT) (**Figure** **1**A,B; Figure S1C, Supporting Information) between aging UCP1^−/−^ and WT mice. Notably, lipid deposition in BAT was increased in aging UCP1^−/−^ mice, compared to age‐matched WT mice (Figure 1C,D). Besides, transmission electron microscopy (TEM) revealed aberrant mitochondrial cristae morphology in BAT of aging UCP1^−/−^ mice (Figure 1D), along with suppressed mitochondrial gene programs (Figure 1E), overall suggesting that UCP1 deficiency impaired mitochondria respiration in BAT.

We also found enhanced hepatic lipid infiltration as well as worsened lipid parameters including nonestesterified fatty acid (NEFA) and total cholesterol (TC) levels in aging UCP1^−/−^ mice compared to controls (Figure S1D–H, Supporting Information), possibly as a result of suppressed BAT functionality. However, despite their BAT dysfunction, aging UCP1^−/−^ mice had sustained glucose metabolism as they performed similarly in glucose tolerance tests (GTT) and insulin tolerance tests (ITT) (Figure 1F,G). Interestingly, the oxygen consumption (VO_2_) and carbon dioxide excretion (VCO_2_) in aging UCP1^−/−^ mice were even increased compared to aging WT mice (Figure 1H,I), without changes in locomotor activity and food intake (Figure S1I,J, Supporting Information).

Taken together, these results suggest that UCP1 deficiency impairs BAT function and lipid metabolism, while sustains glucose homeostasis and increasing energy expenditure in aging UCP1^−/−^mice.

### 2.2 UCP1 Deficiency Sustains Beige Fat Metabolism and Improves Muscle Glycolytic Function and Energy Expenditure in Aging Mice

**Figure 2:**
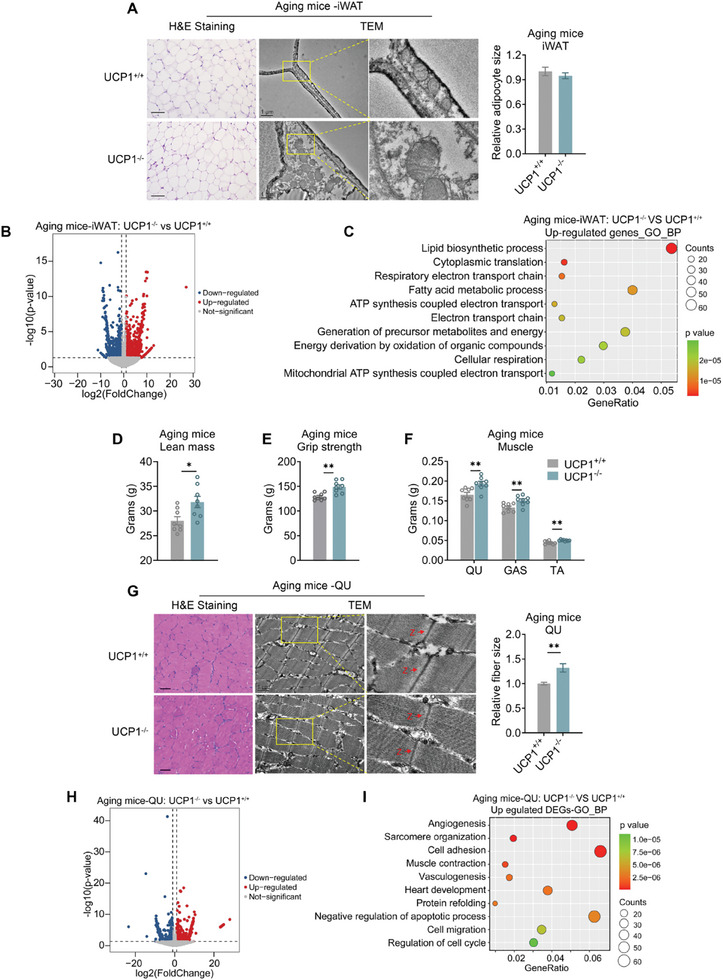
UCP1 deficiency sustains beige fat metabolism and improves muscle glycolytic function in aging mice. A) H&E staining and TEM analysis of iWAT from 12‐month‐old aging UCP1^+/+^ and UCP1^−/−^ mice. B) Volcano plot of differential gene expressions between iWAT from aging UCP1^+/+^ and UCP1^−/−^ mice. C) Representative GO terms of the biological process (BP) categories enriched in upregulated transcripts of iWAT from aging UCP1^−/−^ mice compared to UCP1^+/+^ mice. D–F) Analysis of lean mass D), grip strength E), and weights of skeletal muscles F) from 12‐month‐old aging UCP1^+/+^ and UCP1^−/−^ mice. G) Representative images of H&E staining, relative fiber sizes and TEM analysis of quadriceps (QU) muscle from aging UCP1^+/+^ and UCP1^−/−^ mice. H) Volcano plot of differential gene expressions between QU muscle from aging UCP1^+/+^ and UCP1^−/−^ mice. I) Representative GO terms of the biological process (BP) categories enriched in upregulated transcripts of QU muscle from aging UCP1^−/−^ mice compared to UCP1^+/+^ mice. Data are presented as mean ± SEM and *p < 0.05, **p < 0.01 compared to aging UCP1^+/+^ group. *n* = 8 per group. Scale bars are 45 µm for H&E staining and 1 µm for TEM analysis.

Considering the importance of UCP1 in glucose metabolism and energy expenditure, the comparable metabolic performances between the two genotypes of mice suggest that other metabolic organs might play a compensatory role during aging. Interestingly, unlike in BAT, UCP1 deficiency did not affect lipid deposition in iWAT of aging mice. Furthermore, aging UCP1^−/−^ mice even showed improved mitochondrial morphology in iWAT as assessed by TEM (**Figure** **2**A), accompanied with increased gene programs related to electron transport chain and ATP synthesis as revealed by gene ontology (GO)‐biological process (BP) analysis of RNA‐seq (Figure 2B,C; Table S1, Supporting Information), suggesting that UCP1 deficiency sustained beige fat function and improved mitochondria respiration in iWAT under aging scenario, possibly due to UCP1‐independent thermogenic mechanisms.

Skeletal muscle is another important tissue for energy metabolism. Interestingly, we found that aging UCP1^−/−^ mice showed a significant increase in lean mass and enhancement in grip strength compared to WT mice (Figure 2D,E). Further analysis revealed that aging UCP1^−/−^ mice showed increased quadriceps (QU), gastrocnemius (GAS) and tibialis anterior (TA) muscle weights and larger fiber sizes (Figure 2F; Figure S2A,B, Supporting Information). TEM analysis revealed improved QU muscle structure featuring aligned sarcomere arrangement and organized Z lines in aging UCP1^−/−^ mice, compared to the jagged organization in controls, which is characteristic in aging muscles (Figure 2G). Meanwhile, GO‐BP analysis of RNA‐seq revealed that sarcomere organization and muscle contraction were increased in skeletal muscle of UCP1^−/−^ mice (Figure 2H,I; Table S2, Supporting Information), overall suggesting that skeletal muscles of aging UCP1^−/−^ mice were improved.

**Figure 3:**
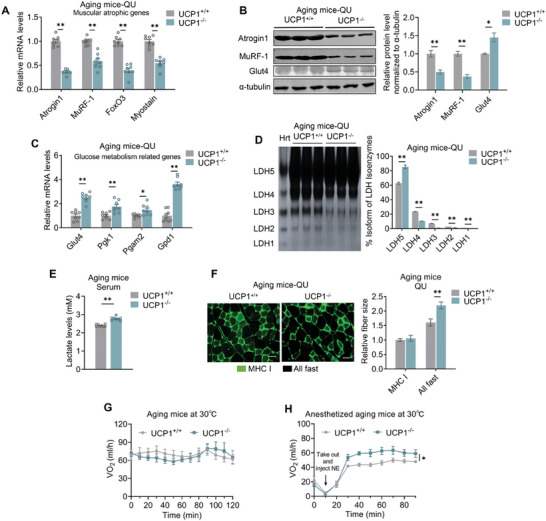
Beige adipocytes crosstalk with myotubes to attenuate atrophy. A,B) Gene A) and protein B) levels of muscle atrophy markers of QU muscle from aging UCP1^+/+^ and UCP1^−/−^ mice. C,D) Gene expression analysis of glucose uptake and glycolysis genes C) and polyacrylamide gel electrophoresis analysis of LDH isoenzyme activity D) of QU muscles from aging UCP1^+/+^ and UCP1^−/−^ mice. E) Lactate levels in serum from aging UCP1^+/+^ and UCP1^−/−^ mice. F) Relative fiber sizes of QU muscle from aging UCP1^+/+^ and UCP1^−/−^ mice stained for MHCI (green). Scale bar is 45 µm. G,H) Oxygen consumption (VO_2)_ of mice housed at 30 °C, without treatment G) or after anesthesia and NE‐injection in GAS and QU H). Data are presented as mean ± SEM and *p < 0.05, **p < 0.01 compared to aging UCP1^+/+^ group. *n* = 8 or 5 per group.

Consistently, we found reduced mRNA and protein levels of muscular atrophic genes, including Atrogin1, MuRF‐1, FoxO3 and Myostatin, in QU, GAS and TA of aging UCP1^−/−^ mice compared to aging WT mice (**Figure** **3**A,B; Figure S3A–D, Supporting Information). Furthermore, qPCR analysis revealed that mRNA levels of genes involved in glucose uptake and glycolysis were significantly higher in muscles from aging UCP1^−/−^ mice than WT mice (Figure 3C; Figure S3E,F, Supporting Information), while mitochondrial gene programs were similar in both genotypes of mice (Figure S3G, Supporting Information), suggesting that UCP1 deficiency may affect glycolytic activity but not oxidative capacity of the muscles. In supporting of this, LDH isoenzyme activity studies indicated that isoenzyme containing LDH‐A, which was enriched in glycolytic myofibers, was increased in aging UCP1^−/−^ mice (Figure 3D; Figure S3H,I, Supporting Information). Moreover, serum lactate levels were increased in aging UCP1^−/−^ mice (Figure 3E). These were not caused by elevated muscle injury in UCP1^−/−^ mice, as shown by comparable creatine kinase activity in both groups of mice (Figure S3J, Supporting Information). Importantly, immunostaining revealed that aging UCP1^−/−^ mice exhibited enlarged sizes of fast type fibers (Figure 3F). Overall, these results suggest that muscle glycolytic function was strengthened in aging UCP1^−/−^ mice.

We next examined whether energy metabolism of skeletal muscle in aging UCP1^−/−^ mice were also enhanced. Aging WT and UCP1^−/−^ mice had similar energy expenditure when housed at thermoneutrality of 30 °C (Figure 3G). These thermoneutral mice were then subjected to anesthesia to shut down both cold‐induced thermogenesis and muscular shivering, and injected norepinephrine (NE) into QU and GAS muscle locally to evaluate their muscle energy metabolism. Compared to aging WT mice, NE muscle injection‐induced oxygen consumption was largely increased in aging UCP1^−/−^ mice (Figure 3H), indicated that aging UCP1^−/−^ mice harbored higher energy metabolic capacity in their skeletal muscles, which may compensate for the defects of BAT function caused by UCP1 deficiency and maintain glucose and energy homeostasis in these mice.

Notably, these changes were specific to aging mice, as young UCP1^−/−^ and UCP1^+/+^ mice showed no differences in body weights, body composition (Figure S4A–C, Supporting Information), grip strength (Figure S4D, Supporting Information), muscle weights (Figure S4E, Supporting Information) and fiber sizes (Figure S4F–H, Supporting Information). In addition, we investigated whether cardiac function was altered in aging UCP1^−/−^ mice. In vivo echocardiography revealed that aging UCP1^−/−^ mice had comparable cardiac function as aging WT mice, as shown by hemodynamics, ventricular function, left ventricular internal diameter of diastolic and systolic, and heart volume, accompanied with similar gene expressions of myocardial hypertrophy and fibrotic markers between the two genotypes of mice (Figure S5A–H, Supporting Information).

Taken together, these data suggest that UCP1 deficiency spared iWAT dysfunction and caused specific enhancements in muscle glycolytic function and energy expenditure in aging mice, which may underline the improved whole‐body energy homeostasis in these mice.

### 2.3 Beige Adipocytes, but not Brown Adipocytes, Engage in Crosstalk with Myotubes to Improve Glucose Metabolism and Mitigate Atrophy

**Figure 4:**
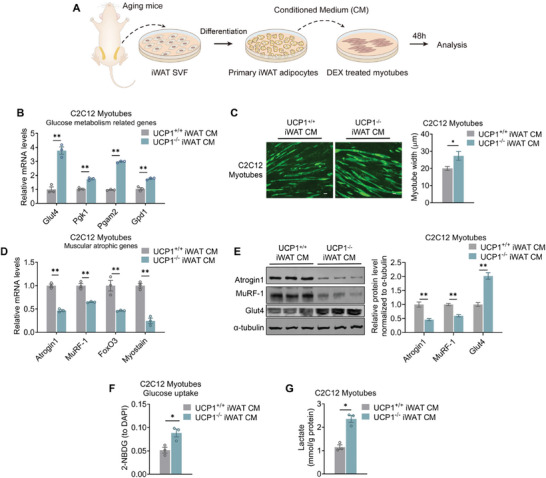
Beige adipocytes crosstalk with myotubes to attenuate atrophy. A) Schematic illustration of co‐culture system of beige adipocytes and myotubes. B) Glucose metabolic gene expression analysis of DEX‐treated C2C12 myotubes cultured with or without conditioned medium (CM) from differentiated primary beige adipocytes of aging UCP1^+/+^ and UCP1^−/−^ mice. C) Representative images of MyHC staining and myotube width of DEX‐treated C2C12 myotubes. D,E) mRNA D) and protein levels E) of muscle atrophic markers of DEX‐treated C2C12 myotubes. F,G) Glucose uptake activity F) and relative lactate levels G) of DEX‐treated C2C12 myotubes cultured with or without CM from differentiated primary beige adipocytes of aging UCP1^+/+^ and UCP1^−/−^ mice. Data are presented as mean ± SEM and *p < 0.05, **p < 0.01 compared to aging UCP1^+/+^ group. Hrt: heart as positive control. *n* = 3 per group. Scale bar is 25 µm. CM: Conditioned medium.

Since UCP1 deficiency predominantly impacts brown or beige adipocytes in mice, there could potentially be an adipose‐muscle crosstalk in play. Thus, we further explored this potential interaction between thermogenic adipocytes and myotubes in vitro. The stromal vascular fraction (SVF) obtained from iWAT and BAT of aging WT and UCP1^−/−^ mice were differentiated into mature adipocytes. Culture medium (CM) from various groups of cells was added into dexamethasone (DEX)‐induced C2C12 myotubes, a cellular model of muscle atrophy, for morphological and molecular analysis (**Figure** **4**A). As shown in Figure 4B–G, compared to atrophic myotubes treated with aging WT iWAT CM, aging UCP1^−/−^ iWAT CM treatment resulted in significant increases in expressions of genes related to glucose uptake and glycolysis, enlargements in myotube sizes, decreases in muscular atrophic protein levels, as well as increased capability of glucose uptake and lactate production in myotubes. In contrast, senescent myotubes treated with aging WT or UCP1^−/−^ BAT CM showed no overt difference (Figure S6A–E, Supporting Information). These data suggested that a specific crosstalk between beige fat and skeletal muscle might be induced in aging UCP1^−/−^ mice.

### 2.4 Creatine Metabolism is Altered in both QU Muscle and iWAT of Aging UCP1 Deficient Mice

**Figure 5:**
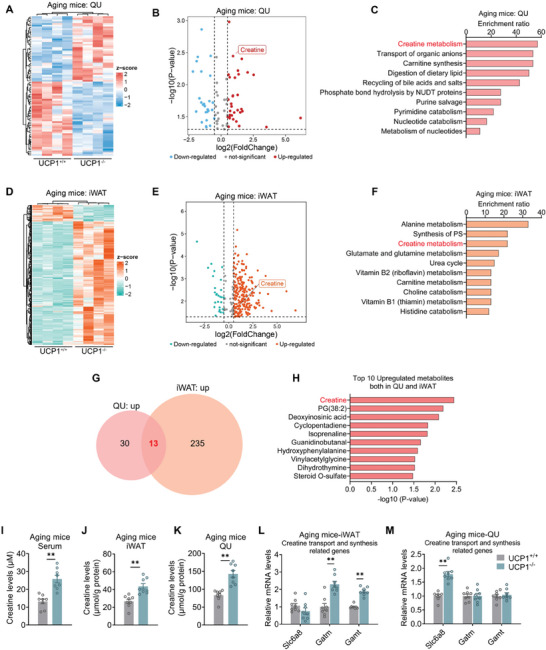
Creatine metabolism is altered in both QU muscle and iWAT of aging UCP1 deficient mice. A,B) Heatmap A) and volcano plot B) of differential metabolites in QU from aging UCP1^−/−^ mice compared to UCP1^+/+^ mice (n = 4). C) Enrichment analysis of up‐regulated metabolites in QU from aging UCP1^−/−^ mice compared to UCP1^+/+^ mice. D,E) Heatmap D) and volcano plot E) of differential metabolites in iWAT from aging UCP1^−/−^ mice compared to UCP1^+/+^ mice. F) Enrichment analysis of up‐regulated metabolites in iWAT from aging UCP1^−/−^ mice compared to UCP1^+/+^ mice. G) Overlapped up‐regulated metabolites from QU and iWAT. H) Top 10 up‐regulated metabolites overlapped from QU and iWAT. I–K) Creatine levels in serum I), iWAT J), and QU K) from aging UCP1^+/+^ and UCP1^−/−^ mice (n = 8). L,M) Gene expression analysis of creatine synthesis and transport in iWAT L) and QU M) from aging UCP1^+/+^ and UCP1^−/−^ mice (n = 8). Data are presented as mean ± SEM and *p < 0.05, **p < 0.01 compared to control group. n = 4 or 8 per group.

Next, we sought to understand how iWAT of UCP1^−/−^ aging mice cross‐talked with and improved skeletal muscle. Metabolites have been well recognized as vital mediators for tissue communications. Thus, we performed a high‐resolution mass spectrometry‐based untargeted metabolomics using iWAT and QU muscle of aging WT and UCP1^−/−^ mice. The heatmap and volcano plot of differential metabolites were presented, and metabolite set enrichment analysis (MSEA) was performed in both iWAT and QU muscle. Interestingly, MSEA of the upregulated metabolites revealed creatine metabolism as the common metabolic pathway enriched in both tissues of aging UCP1^−/−^ mice (**Figure** **5**A–F; Figure S5A–C, Tables S3 and S4, Supporting Information). We further examined the characteristic metabolites that exhibited significant upregulation in both iWAT and QU in UCP1^−/−^ versus WT mice, which yielded 13 upregulated metabolites after overlapping (Figure 5G; Figure S7C; Table S5, Supporting Information). Among them, creatine is the top‐ranked upregulated metabolite (Figure 5H), which is consistent with the KEGG analysis. Indeed, biochemical analysis confirmed that creatine levels in serum, iWAT and skeletal muscles including QU, GAS and TA muscle were all increased in aging UCP1^−/−^ mice compared to WT mice (Figure 5I–K; Figure S7D,E, Supporting Information).

To understand the source of increased creatine in both iWAT and skeletal muscle, we further investigated the expression of creatine biosynthesis and transport genes in these tissues from aging WT and UCP1^−/−^ mice. Creatine can be synthesized through a two‐step enzymatic reaction involving glycine amidinotransferase (*Gatm*) and guanidinoacetate N‐methyltransferase (*Gamt*).^[^ ^23^ ^]^ Additionally, creatine circulates in the blood and is transported into creatine‐demanding tissue mediated by the creatine transporter SLC6A8. Interestingly, *Gatm* and *Gamt* were specifically increased in iWAT while the creatine uptake transporter gene *Slc6a8* was specifically increased in skeletal muscle of UCP1^−/−^ mice (Figure 5L,M; Figure S7F,G, Supporting Information), without changes in liver (Figure S7H,I, Supporting Information), suggesting that creatine may be synthesized in iWAT, and then transported to and utilized by skeletal muscle. Interestingly, we also found that the expression level of Slc6a8 in skeletal muscle was increased in aging mice compared to young mice (Figure S7J,K, Supporting Information), suggesting that the ability of creatine uptake was increased in muscle of aging mice, which might be a compensatory consequence of UCP1 impairment in thermogenic fat.

### 2.5 Creatine‐Mediated Crosstalk between iWAT and Skeletal Muscle of Aging UCP1^−/−^ Mice Determines Increased Muscle Glycolytic Functionality and Suppressed Atrophy

**Figure 6:**
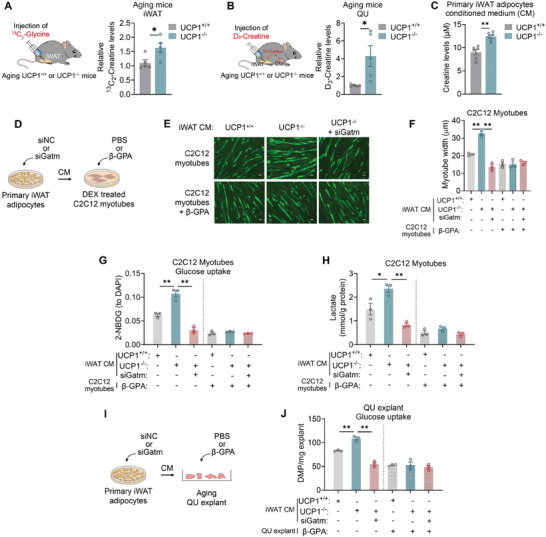
Creatine‐mediated crosstalk between iWAT and skeletal muscle determines muscle glycolytic functionality in vitro and *ex vivo*. A) Metabolic flux analysis of ^13^C_2_‐Glycine injected in iWAT and detection of labeled creatine in iWAT. B) Metabolic flux analysis of D_3_‐Creatine injected in iWAT and detection of labeled creatine in QU muscle. C) Creatine levels in conditioned medium from primary iWAT adipocytes. D) Schematic illustration of analysis of creatine‐mediated crosstalk between primary beige adipocytes and C2C12 myotubes. E–H) Representative images of MyHC staining E), myotube width F), glucose uptake activity G) and relative lactate levels H) of DEX‐treated C2C12 myotubes treated with iWAT culture medium (CM). I) Schematic illustration of analysis of creatine‐mediated crosstalk between primary beige adipocytes and QU muscle explant. J) Glucose uptake activity of QU muscle explant treated with iWAT CM. Data are presented as mean ± SEM and *p < 0.05, **p < 0.01 compared to control group. *n* = 5 or 3 per group.

Our data suggested that UCP1 deficiency in aging mice induces the synthesis and secretion of creatine in iWAT, and stimulates creatine uptake in skeletal muscle. To further clarify interplay between beige fat and skeletal muscle via creatine during aging, we conducted in vivo metabolic flux analysis in aging WT and UCP1^−/−^ mice. After local injection of ^13^C_2_‐glycine, a direct precursor to creatine, into iWAT of these aging mice, we monitored its metabolic fate using LC/MS and found increased accumulation of ^13^C_2_‐creatine in iWAT from aging UCP1^−/−^ mice (**Figure** **6**A), suggesting increased creatine synthesis in these mice compared to aging WT mice. In addition, after local injection of stable isotope labeled creatine (D_3_‐creatine) into iWAT of aging WT and UCP1^−/−^ mice, we tracked its accumulation in QU muscle via LC/MS and discovered that aging UCP1^−/−^ mice took up significantly more D_3_‐creatine in their QU muscle than aging WT mice (Figure 6B).

We further assessed the creatine levels in conditioned medium from primary iWAT adipocytes and found that primary UCP1^−/−^ iWAT CM featured significantly higher creatine level when compared to the primary WT iWAT CM (Figure 6C). Moreover, we used siRNA targeting *Gatm*, the rate‐limiting enzyme in creatine biosynthesis, in primary beige adipocytes from aging UCP1^−/−^ mice. The culture medium was collected and used to incubate DEX‐induced atrophic myotubes in the presence of PBS or β‐GPA, a well‐established creatine analog to inhibit creatine transport^[^ ^14^, ^24^, ^25^ ^]^ (Figure 6D). Interestingly, CM of UCP1^−/−^ primary beige adipocytes induced myotube hypertrophy and improved glycolytic capability, as shown by myotube immunofluorescence (Figure 6E,F), glucose uptake assay (Figure 6G) and lactate levels in myotubes (Figure 6H), while both Gatm knockdown in aging UCP1^−/−^ beige adipocytes or β‐GPA addition to myotubes completely blunted these effects. Besides, similar results were observed in *ex vivo* experiments. Incubation of aging QU muscle explants in CM of UCP1^−/−^ primary beige adipocytes increased glucose uptake in muscle explants, which was blocked by siGatm in beige adipocytes or β‐GPA treatment in QU muscle explants (Figure 6I,J). These data suggested that creatine‐mediated crosstalk between iWAT and skeletal muscle of aging UCP1^−/−^ mice determines increased muscle glycolytic functionality.

### 2.6 β‐GPA Treatment Deteriorates Glucose Homeostasis and Skeletal Muscle Function in Aging UCP1^−/−^ Mice

**Figure 7:**
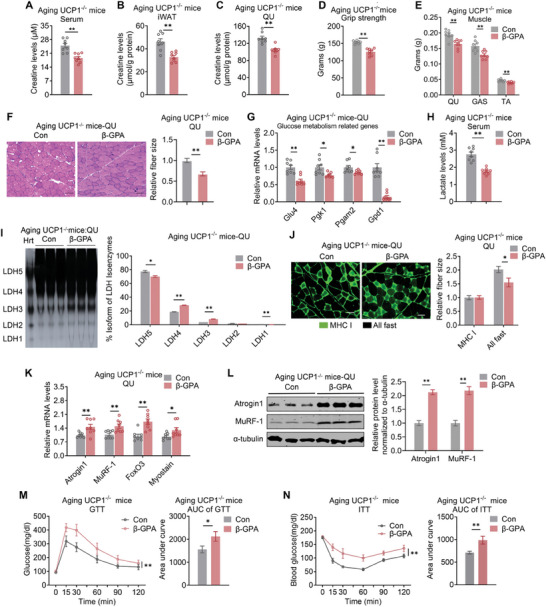
β‐guanidinopropionic acid (β‐GPA) treatment deteriorates skeletal muscle function in aging UCP1^−/−^ mice. A–C) Creatine levels in serum A), iWAT B), and QU C) from aging UCP1^−/−^ mice treated with β‐GPA or vehicle. D,E) Grip strength analysis D) and weights of skeletal muscle E) of aging UCP1^−/−^ mice treated with β‐GPA or vehicle. F) Representative images of H&E staining and relative fiber sizes of QU from aging UCP1^−/−^ mice treated with β‐GPA or vehicle. Scale bar is 45 µm. G) Gene expression analysis of glucose uptake and glycolysis of QU from aging UCP1^−/−^ mice treated with β‐GPA or vehicle. H) Lactate levels in serum from aging UCP1^−/−^ mice treated with β‐GPA or vehicle. I) Polyacrylamide gel electrophoresis analysis of LDH isoenzyme activity of QU from aging UCP1^−/−^ mice treated with β‐GPA or vehicle. J) Relative fiber sizes of QU muscle stained for MHCI (green) from aging UCP1^−/−^ treated with β‐GPA or vehicle. Scale bar is 45 µm. K,L) Gene K) and protein L) expression analysis of muscle atrophy markers of QU from aging UCP1^−/−^ mice treated with β‐GPA or vehicle. M,N) GTT M) and ITT N) assay with AUC quantification of aging UCP1^−/−^ control or β‐GPA injected mice. Mice were injected intraperitoneally with glucose at 1 g kg^−1^ body weight for GTT and insulin at 0.75 U kg^−1^ body weight for ITT. Data are presented as mean ± SEM and *p < 0.05, **p < 0.01 compared to control group. β‐GPA: β‐guanidinopropionic acid; Hrt: heart as positive control. *n* = 8 per group.

Furthermore, to investigate whether creatine mediates the crosstalk between iWAT and skeletal muscle in aging UCP1^−/−^ mice in vivo, we treated aging UCP1^−/−^ mice with either vehicle or β‐GPA to shut down creatine transport. Compared to controls, the creatine levels were decreased in serum, iWAT and skeletal muscles including QU, GAS and TA of aging UCP1^−/−^ mice upon β‐GPA treatment (**Figure** **7**A–C; Figure S8A,B, Supporting Information). Notably, aging UCP1^−/−^ mice treated with β‐GPA exhibited significantly lower grip strength (Figure 7D), reduced muscle weights and fiber sizes (Figure 7E,F; Figure S8C,D, Supporting Information) compared to control group. In addition, β‐GPA treatment also reversed the upregulation of glucose uptake and glycolysis genes in skeletal muscles of aging UCP1^−/−^ mice, while did not alter mitochondrial genes (Figure 7G; Figure S8E–G, Supporting Information). Consistent with that, serum lactate levels were lower in β‐GPA treated aging UCP1^−/−^ mice (Figure 7H), along with a consistent shift in LDH isoenzyme activity (Figure 7I). Moreover, β‐GPA treated aging UCP1^−/−^ mice exhibited smaller sizes of fast type muscle fibers as shown by immunofluorescence staining (Figure 7J). Meanwhile, β‐GPA treatment increased the expression of muscular atrophic genes and proteins in skeletal muscles (Figure 7K,L; Figure S8H,I, Supporting Information). Of note, though 6‐week β‐GPA treatment did not influence body weights or fat mass of aging UCP1^−/−^ mice, they displayed impaired glucose metabolism as shown in GTT and ITT analysis (Figure 7M,N; Figure S8J–M, Supporting Information). Overall, these results demonstrate that glucose homeostasis and the enhanced muscle function in aging UCP1^−/−^ mice were abolished upon β‐GPA treatment and subsequent reduction in creatine levels, suggesting that creatine mediates the enhancement in skeletal muscle function that compensates for the decline of thermogenic fat function and maintains glucose homeostasis during aging.

### 2.7 UCP1 Inhibitor α‐CD Administration Leads to an Improved Muscle Glycolytic Functionality in Aging Mice

**Figure 8:**
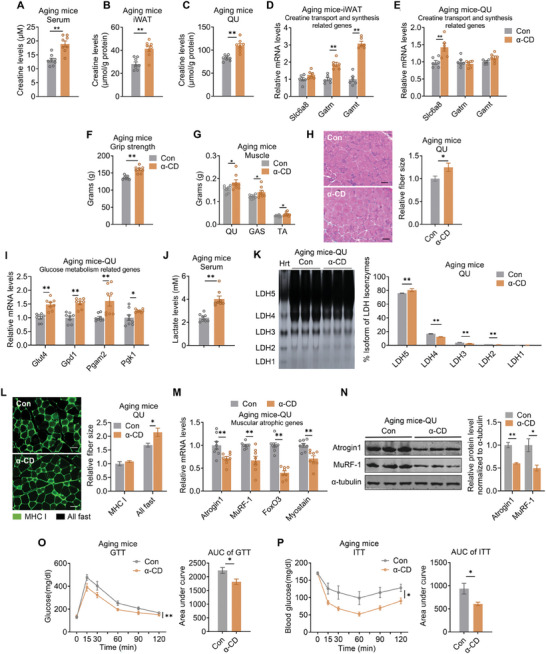
UCP1 inhibitor α‐CD administration leads to improved muscle glycolytic functionality and glucose metabolism in aging mice. A–C) Creatine levels in serum A), iWAT B), and QU C) from aging mice treated with α‐CD or vehicle. D,E) Gene expression analysis of creatine synthesis and transport in iWAT D) and QU E) from aging mice treated with α‐CD or vehicle. F,G) Grip strength analysis F) and weight of QU, GAS and TA G) of aging mice treated with α‐CD or vehicle. H) Representative images of H&E staining and relative fiber sizes of QU from aging mice treated with α‐CD or vehicle. Scale bar is 45 µm. I) Gene expression analysis of glucose uptake and glycolysis genes of QU from aging mice treated with α‐CD or vehicle. J) Lactate levels in serum from aging mice treated with α‐CD or vehicle. K) Polyacrylamide gel electrophoresis analysis of LDH isoenzyme activity of QU from aging mice treated with α‐CD or vehicle. L) Relative fiber sizes of QU muscle from aging mice treated with α‐CD or vehicle stained for MHCI (green). Scale bar is 45 µm. M,N) qPCR M) and protein N) analysis of muscle atrophy markers in QU from aging mice treated with α‐CD or vehicle. O,P) GTT O) and ITT P) assay with AUC quantification of aging mice treated with α‐CD or vehicle. Mice were injected intraperitoneally with glucose at 1.5 g kg^−1^ body weight for GTT, and insulin at 0.75 U kg^−1^ body weight for ITT. Data are presented as mean ± SEM and *p < 0.05, **p < 0.01 compared to aging group. α‐CD: α‐cyclodextrin. Hrt: heart as positive control. n = 8 per group. n = 8 per group.

We further investigated whether pharmacological inhibition of UCP1 activity in aging mice could mimic phenotypes of aging UCP1^−/−^ mice. UCP1 is activated by long‐chain fatty acids. α‐cyclodextrin (α‐CD), a non‐toxic cyclic oligosaccharide formed by six glucose units, competes with long‐chain fatty acids for UCP1 binding and thus can block UCP1 activation.^[^ ^26^ ^]^ Therefore, we administered α‐CD intraperitoneally (i.p.) to aging mice to assess the effect of UCP1 inactivation on compensatory attenuation of aging‐associated skeletal muscular atrophy. Indeed, we observed similar phenotypes in α‐CD treated aging mice as aging UCP1^−/−^ mice. Creatine levels in serum, iWAT and skeletal muscle were higher in α‐CD group than those in the control group (**Figure** **8**A–C; Figure S9A,B, Supporting Information), with increased expression of creatine biosynthetic genes in iWAT and creatine uptake gene *Slc6a8* in skeletal muscles (Figure 8D,E; Figure S9C,D, Supporting Information). Meanwhile, α‐CD treatment mildly increased mitochondrial genes in iWAT (Figure S9E, Supporting Information), while the grip strength of aging mice treated with α‐CD were significantly increased (Figure 8F), consistent with increased weights and fiber sizes of skeletal muscles (Figure 8G,H; Figure S9F,G, Supporting Information). Detailed analysis revealed that α‐CD treatment increased the expression of glucose uptake and glycolysis genes, but not mitochondrial genes in skeletal muscle (Figure 8I; Figure S9H–J, Supporting Information). Consistently, α‐CD group also featured higher serum lactate contents, LDH isoenzyme shifts in muscles (Figure 8J,K), larger size of fast type fibers (Figure 8L), decreased muscular atrophic genes (Figure 8M,N; Figure S9K–N, Supporting Information), all suggested enhanced muscle glycolytic function in α‐CD treated mice compared to the control group. Of note, the α‐CD group showed improved glucose homeostasis as indicated by better performances in GTT and ITT compared with control group (Figure 8O,P). However, α‐CD treatment did not influence energy expenditure, locomotor activity, food intake, body weight, body composition or fat weight in aging mice (Figure S10A–H, Supporting Information), with mild effects on BAT (Figure S10I, Supporting Information). Besides, we found that α‐CD treatment in aging mice did not cause defects in liver, as both groups showed similar liver weights, hepatic triglyceride levels and morphology, suggesting the safeness of α‐CD application in aging mice for metabolic improvements (Figure S10J–L, Supporting Information). Overall, our findings suggest that i.p. administration of α‐CD in aging mice alleviates aging‐associated sarcopenia and improves glucose homeostasis in vivo.

## 3 Discussion

UCP1 is the most well‐known thermogenic effector and a key regulator of cold‐mediated thermogenesis.^[^ ^3^, ^4^ ^]^ It has been widely accepted that UCP1 primarily mediates the “anti‐obesity” and “anti‐diabetic” actions of brown and beige fat. Thus, it is surprising that inactivation of UCP1 did not potentiate diet‐induced obesity. Genetic studies in rodent models have revealed a discrepancy in the metabolic phenotypes between brown/beige fat‐deficient mice and UCP1‐deficient mice. For instance, mice deficient in BAT or iWAT exhibit obese phenotypes under ambient temperature conditions.^[^ ^27^, ^28^, ^29^ ^]^ In contrast, UCP1‐deficient mice do not develop obesity under the same conditions and only become obese when kept under thermoneutrality.^[^ ^22^, ^30^ ^]^ This apparent contradiction in the metabolic phenotypes between these mouse models implies the existence of UCP1‐independent mechanisms through which brown and/or beige fat contribute to the regulation of whole‐body energy homeostasis. Among these UCP1‐independent pathways, creatine futile cycle, a cycling of creatine and phospho‐creatine fueled by mitochondrial ATP production and consumption, has been shown to play an indispensable role in energy metabolism.^[^ ^14^, ^31^ ^]^ We demonstrate that creatine derived from adipose tissue is essential for improving skeletal muscle function during aging. Interestingly, this improvement was not significant in young mice, suggesting an age‐specific effect. As there exist multiple UCP1‐independent thermogenic pathways, it is possible that other futile cycles in thermogenic fat are intact to compensate for UCP1 loss at young age, while futile creatine cycle becomes dominant in aging.

Aging is featured of a functional decline across organs as well as impaired tissue crosstalk, which impacts systemic energy homeostasis. Systemic organ‐specific temporal analysis showed that adipose tissues show signs of aging earlier than muscle functionality decline,^[^ ^32^, ^33^ ^]^ including a programmed loss of thermogenic genes in murine subcutaneous adipose tissue.^[^ ^11^, ^12^ ^]^ Meanwhile, aging in skeletal muscle is associated with muscle atrophy or sarcopenia, which often occurs in the later stage of aging and severely affects human health and life quality.^[^ ^34^ ^]^ This indicated a possible link between adipose tissues aging and muscle deterioration. Although crosstalk between adipose tissues and skeletal muscle have been reported, little is known about how these two tissues coordinate metabolic maintenance and how accelerated aging in adipose tissue affects muscle functionality. It has long been noted that aging often leads to obesity and metabolic dysfunction. However, to date, the metabolic changes among metabolic organs observed late in life have not yet been fully characterized. In this study, we demonstrate that UCP1 deficiency in brown fat leads to a compensatory increase in creatine synthesis in the beige adipose tissue of aging mice. UCP1 decouples proton leakage from ATP synthesis. We found enhanced mitochondrial ATP‐synthesis coupled electron transport in aging UCP1 KO mice compared to WT mice, which could be result of the absence of UCP1 and decreased UCP1‐mediated proton gradient dissipation. This increase in ATP production could in turn drive creatine futile cycle, which consumes excessive ATP. Meanwhile, enhanced creatine can then be secreted into circulation and taken up by skeletal muscle to resist aging‐induced muscular atrophy and maintain glucose homeostasis. Interestingly, it has been long recognized that practicing sport in cold environments mitigates sports performances of athletes.^[^ ^35^ ^]^ Moreover, it is observed that augmented diurnal temperature fluctuation aggravates muscular atrophy in elders,^[^ ^36^ ^]^ highlighting a potential relationship between thermogenic fat activation and impaired muscle functionality. Furthermore, human studies revealed that serum concentration of ATPase Inhibitory Factor 1 was negatively and significantly correlated with oxygen consumption and exercise capacity.^[^ ^37^ ^]^ This is in line with our observation that UCP1 ablation increases ATP production and creatine cycle, which promote muscle function. Thus, it is possible that similar compensatory mechanisms involving creatine metabolism and muscle function also exist in humans. Our findings provide insights into the tissue crosstalk mechanisms underlying the chronobiology of aging and the maintenance of metabolic homeostatic during aging.

Aside from creatine cycle, other futile cycles have been reported to play compensatory roles for UCP1 independent thermogenesis in adipose tissues, such as lipid futile cycle and Ca^2+^/ATPase (SERCA) cycle.^[^ ^4^ ^]^ Specifically, the futile ATP‐consuming triglyceride/fatty acid cycle serves as a major contributor to cellular heat production in thermogenic adipocytes lacking UCP1.^[^ ^38^, ^39^ ^]^ Meanwhile, Ca^2+^ futile cycle depends on ATP‐dependent Ca^2+^ cycling via SERCA2b and Ca^2+^ release channel RyR2 and IP3R, providing another UCP1 independent mechanism for thermogenesis.^[^ ^40^, ^41^, ^42^ ^]^ For aging scenario, it has been recently reported that β3‐adrenergic agonist CL316243 treatment in aging mice increased lipogenesis and lipid turnover in iWAT, suggesting the activation of the futile substrate cycle of lipolysis and re‐esterification.^[^ ^43^ ^]^ Besides, sarcoplasmic reticulum‐related factors were also found to be increased in skeletal muscle of aging mice.^[^ ^44^ ^]^ These studies suggested that other compensatory mechanisms may also be involved in the regulation of adipose tissue and skeletal muscle crosstalk upon UCP1 deficiency during aging, which needs further investigation.

Sarcopenia, characterized by the progressive loss of skeletal muscle mass and function, is caused by altered neuromuscular function, oxidative stress, systemic inflammation, mitochondrial dysfunction and hormonal imbalance, significantly impacts health outcomes in older adults.^[^ ^34^ ^]^ Current management strategies primarily involve resistance exercise and nutritional interventions, especially the recommendation of combination of resistance training and increased protein and calorie intake.^[^ ^45^, ^46^ ^]^ Furthermore, pharmaceutical development at various stages of clinical trials for sarcopenia intervention include myostatin inhibitors, selective androgen receptor modulators, ghrelin receptor agonists, mesenchymal stem cell therapy and follistatin gene therapy, while dietary patterns, cultural contexts and distinct drug responses of human population may limit their effectiveness.^[^ ^47^, ^48^ ^]^ Intriguingly, thermogenic fat can crosstalk with muscle, which rendered adipose tissue a promising intervention target for muscle atrophy. Of note, creatine is widely used to enhance muscle mass and strength in athletes, as well as improve musculoskeletal performance in clinical populations and elders.^[^ ^49^, ^50^ ^]^ However, chronic and excess creatine supplementation may cause adverse effects such as renal dysfunction and rhabdomyolysis.^[^ ^51^, ^52^, ^53^, ^54^, ^55^ ^]^ In the current study, we found compensatory mechanism between adipose tissue and skeletal muscle via creatine metabolism, which involves physiological changes that happens naturally during aging in UCP1 KO mice without adverse effects, thus might be developed as a safe and potential therapeutic strategy against aging‐associated sarcopenia. Indeed, we found that dietary supplementation of UCP1 inhibitor α‐cyclodextrin (α‐CD) in aging mice could enhance the synthesis and secretion of creatine from iWAT, which in turn increase crosstalk between iWAT and muscle via creatine and ultimately decelerate the decline of muscle function associated with aging. α‐CD is derived from starch and is widely used in the food industry, where it enhances the flavor and stability of various food products.^[^ ^56^ ^]^ In addition, α‐CD, as one kind of resistant starch, has been shown to modify gut microbiota, reduce fat accumulation in high fat diet‐fed obese mice and prevent atherosclerosis of ApoE deficient mice,^[^ ^57^, ^58^, ^59^ ^]^ Overall suggesting α‐CD as a safe and effective supplement in combating obesity and aging associated metabolic diseases. Future efforts are warranted to comprehensively screen the secreted mediators from thermogenic fat with potential muscle‐improving properties, which could serve as potential adipose tissue targets to mitigate aging‐related muscle atrophy.

The key finding of this work is the crosstalk between beige fat and skeletal muscle in the aging scenario via creatine metabolism. Future research exploring other potential mediators and mechanisms of this crosstalk would provide more comprehensive understanding. Additionally, it would be intriguing to develop therapeutic strategies combat metabolic dysfunction associated with aging and delve deeper into the underlying mechanisms. For instance, dietary supplementation with α‐CD, which has been shown to prevent obesity in mice by altering the intestinal microbiota,^[^ ^57^ ^]^ warrants further investigations in aging mice and elderly populations. This could potentially provide a novel approach to managing aging‐associated metabolic disorders. It has to be noticed that α‐CD is shown to sequester fatty acids and inhibit UCP1 activity in BAT mitoplasts in vitro,^[^ ^26^ ^]^ while it's in vivo function on thermogenic fat warrants future exploration. It is also reported that α‐CD enhanced myoblasts fusion and muscle differentiation in vitro.^[^ ^60^ ^]^ Besides, previous in vivo studies of α‐CD employed oral administration, which highlighted contributions of α‐CD in preventing dietary fat absorption in both mice and human, possibly by improving the composition of gut microbiota and promoting intestinal GLP‐1 excretion.^[^ ^57^, ^58^, ^59^, ^61^, ^62^, ^63^ ^]^ In this study, we used i.p injection to administrate α‐CD in mice, which minimized the possible effects of α‐CD on intestine. However, we could not fully exclude the possibility that α‐CD may also contribute to the observed phenotypes in aging mice through other reported mechanisms.

## 4 Conclusion

**Figure 9:**
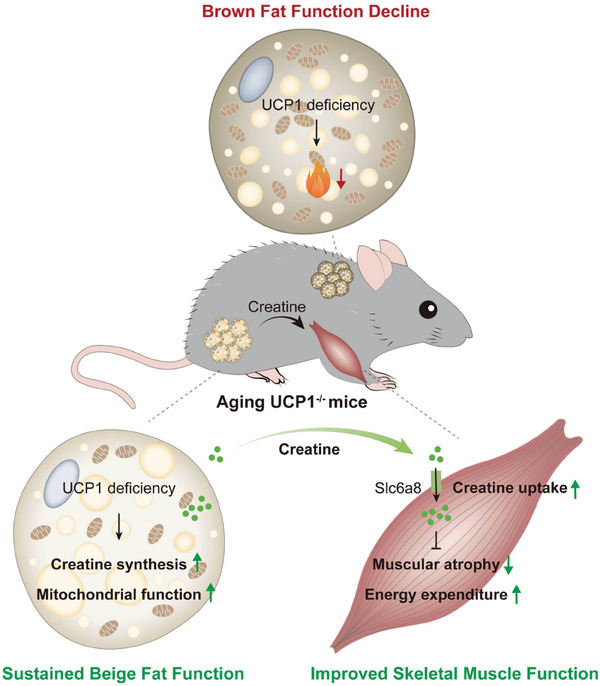
Schematic diagram of the metabolic homeostatic regulation in aging UCP1^−/−^ mice. In aging UCP1^−/−^ mice, brown fat function declines, while beige fat‐specific creatine biosynthesis is increased to sustain its mitochondrial function. Besides, the enhanced secreted creatine from beige fat is taken up by skeletal muscle and compensatory improvements in muscle glycolytic function, thereby increasing energy metabolism.

Whole‐body energy homeostasis is strictly maintained through complicated inter‐tissue crosstalk. In the present study, we discovered an important role for beige adipocyte‐specific creatine biosynthesis in maintaining skeletal muscle function of aging mice when thermogenic effector UCP1 was deficient (**Figure** **9**). Our findings reveal a novel pathway, triggered by UCP1 deficiency in beige fat and centered on creatine metabolism, that facilitates communication between beige adipocytes and myotubes to enhance muscle functionality, thus compensates for whole‐body glucose dysregulation and energy expenditure decline during aging. Furthermore, we found that α‐CD, which inhibits UCP1 activity, can improve iWAT and skeletal muscle functions and alleviate insulin resistance in aging mice, suggesting a potential therapeutic strategy against aging‐associated sarcopenia and insulin resistance.

## 5 Experimental Section

### Animals

Animal experiments were performed in accordance with guidelines of the Animal Care and the Animal Ethics Committee of East China Normal University (m20221206). UCP1^+/+^ and UCP1^−/−^ mice were purchased from Jackson Laboratory and maintained in a humidity and temperature‐controlled facility with free access to water and food. Young (3‐month‐old) and aging (12‐month‐old) male mice were used in the study. Aging mice treated with α‐CD (4 mg kg^−1^ body weight, Sigma, C4642) or β‐GPA (0.4 g kg^−1^ body weight, Sigma, G6878) were injected intraperitoneally three times a week for 6 weeks. The corresponding control mice were injected with the same amount of 0.9% normal saline. Fat mass and lean mass were measured by AccuFat‐1050 MRI system (Meg‐Med). Mice were sacrificed under 1% sodium pentobarbital solution (50 mg kg^−1^ body weight, i.p.). Blood and tissue samples were collected for further analysis.

### Tissue Explant Culture

QU muscle were obtained from aging WT or UCP1^−/−^ mice. Tissue explants were prepared as previously described.^[^ ^64^, ^65^, ^66^, ^67^ ^]^ Briefly, QU muscle were finely diced and placed in DMEM at 37 °C for 1 h. 50 mg tissues were then briefly dried on sterile filter paper and immediately transferred to 24‐well tissue culture plates. Dissected QU tissues were incubated in DMEM (with 100 U ml^−1^ penicillin G and 100 µg ml^−1^ streptomycin) for further treatment.

### Primary Adipocyte Isolation and Differentiation

Stromal vascular fraction (SVF) from mice iWAT and BAT were separated as described previously.^[^ ^68^ ^]^ Briefly, mice iWAT and BAT were isolated, finely minced, and subjected to collagenase digestion for 25 min at 37 °C. The SVF was pelleted and resuspended in DMEM medium containing 20% FBS, 1% penicillin, and 1% streptomycin, and culture medium was changed daily. The SVFs were cultured and differentiated following a standard protocol. Briefly, after reaching confluence (day 0), differentiation was initiated by adding differentiation medium containing 5 µg ml^−1^ insulin (Eli Lilly, HI0240), 0.5 mM isobutylmethylxanthine (Sigma, I7018), 1 µM dexamethasone (Sigma, D4902), 1 nM T3 (Sigma, T2877), and 1 µM rosiglitazone (Sigma, R2408). After 2 days, the medium was replaced with insulin and T3. Medium was changed every 2 days until day 8. The culture medium was collected at day 8 and referred to as primary iWAT and BAT conditioned medium.

### C2C12 Cell Culture and Differentiation

Murine C2C12 myoblasts were purchased from American Type Culture Collection (ATCC) maintained in proliferation medium, consisting of Dulbecco's modified Eagle's medium (DMEM) (Invitrogen), 10% fetal bovine serum (HyClone, Thermo Scientific), and 1% penicillin/streptomycin (P/S) (Gibco, Invitrogen). C2C12 myoblasts were differentiated with low‐serum differentiation medium, consisting of DMEM with 2% horse serum (Thermo Fisher Scientific) and 1% P/S at 37 °C, 5% CO_2_. After 4 days of differentiation, 50 µM dexamethasone was added for 48 h to induce muscular atrophy in vitro, follow by treatment of conditioned medium from differentiated primary brown or beige adipocytes from BAT or iWAT of aging WT and UCP1^−/−^ mice for 48 h. Then, C2C12 myotubes were stained with MHCI and the staining images were captured with fluorescence microscope. Myotube width were quantified from 10 random fields of view using Image‐Pro Plus 6.0 software. Results were shown as the fold change in diameter relative to the control group.

### Grip Strength Test

A forelimb grip strength test was performed in mice using a commercial digital grip strength meter (BIOSEB, BIO‐G53). Mice held by the tail were gently allowed to grasp a wire grid with fore paws. The mice were then gently pulled by the tail until they released their grip. The peak force applied by the limbs of the mouse was recorded in grams. Each mouse was allowed to do ten trials. The average of the three largest values was used for statistical analysis.

### Glucose Tolerance Test (GTT) and Insulin Tolerance Test (ITT) Analysis

For GTT, mice were fasted for 16 h and subsequently injected intraperitoneally with glucose (Sigma, 47 829) dissolved in saline at 1.5 g kg^−1^ body weight or 1 g kg^−1^ body weight. For ITT, mice were fasted for 4 h and injected intraperitoneally with insulin (Sigma, I9278) at 0.75 U kg^−1^ body weight.^[^ ^69^ ^]^ Venous blood was collected from the tail for measurement using ACCU‐CHEK Active (Roche) at t = 0 min, and at t = 15, 30, 60, 90 and 120 min, respectively, after glucose or insulin injection. Area under the curve (AUC) was determined by Graphpad as described.^[^ ^70^ ^]^

### Comprehensive Lab Animal Monitoring System (CLAMS) Analysis

Mice were housed individually in metabolic cages with free access to food and water using a Comprehensive Lab Animal Monitoring System (CLAMS) (Columbus Instruments). The system temperature was set at 24 °C, and on a 12 h standard light‐dark cycle (light 6:00‐18:00, dark 18:00‐6:00). Oxygen and carbon dioxide consumption were recorded by indirect calorimetry method after 48 h of adaptation period. Total locomotor activity was analyzed by infrared beam (Opto‐Varimex mini, Columbus Instruments). The VO_2_ and VCO_2_ data were normalized to per mouse using ANCOVA analysis.^[^ ^71^ ^]^

For measuring metabolic capacity of skeletal muscle, mice were anesthetized and the baseline‐ and NE (1 mg kg^−1^)‐induced O_2_ consumptions were monitored at 30 °C in a CLAMS system chamber. NE was locally injected into the QU and GAS of anesthetized mice.^[^ ^72^ ^]^ Prior to the experiment, mice were acclimated in the chamber for 24 h.

### Glucose Uptake Assay

Glucose uptake for QU muscle explant was measured by the uptake of 2‐deoxy‐d‐[14C] glucose (PerkinElmer, NEC720A250UC). Briefly, QU muscle explant were treated with 0.1 µCi 2‐deoxy‐d‐[14C] glucose and harvested 1 h later. Samples were immediately digested in NaOH at 60 °C for 2 h followed by addition of HCL and centrifugation. The radioactivity of supernatant was measured by a Liquid Scintillation Counter (Tri‐Carb, 4910TR). The glucose uptake was normalized by protein concentrations as quantified by BCA method.

For glucose uptake assay of C2C12 myotubes, cells were washed with PBS and incubated with 100 µM 2‐NBDG (MedChemExpress, HY‐116215), a fluorescently‐labeled deoxyglucose analog, in medium at 37 °C for 30 min. Cells were washed by PBS for three times and then fixed with 1% PFA. The uptake of 2‐NBDG was measured by a SpectraMax M2 microplate fluorometer (Molecular Devices) and the fluorescent intensities of 2‐NBDG uptake were normalized relative to DAPI fluorescent intensities.

### Tissue Collection

At the end of each experiment, mice were weighed and humanely sacrificed. Blood samples were collected and placed overnight at 4 °C. Subsequently, the serum was separated and stored at −80 °C. Skeletal muscles, iWAT and other tissues were weighed and stored at −80 °C. For histological analysis, muscle tissues were embedded in optimal cutting temperature (O.C.T.) compound (SAKURA Tissue‐Tek, 4583), immediately frozen in isopentane cooled in liquid nitrogen and stored at −80 °C. Adipose tissues were fixed in 10% neutral buffered formalin overnight and embedded in paraffin according to standard procedures.

### Transmission Electron Microscopy (TEM) Analysis

Adipose tissues and QU muscle (1 mm^3^) were fixed with 2.5% glutaraldehyde at 4 °C and post‐fixed with 1% OsO_4_. The materials were then washed three times with 0.1 M PBS and dehydrated in a graded series of ethanol. After penetration with 100% acetone, the materials were embedded with Epon 812 and successively polymerized at 37 °C for 18 h, 48 °C for 24 h and at 60 °C for 48 h. The embedded samples were finally ultrathin‐sectioned for 70 nm and stained with uranyl acetae and lead citrate for transmission electron microscopic (JEOL, JEM2100) observation and photography.

### Gene Expression Analysis

Total RNA was extracted from tissues with Trizol (Takara, 9109). 1 µg total RNA was reversely transcribed into cDNA with PrimeScript RT reagent Kit (Takara, PR047Q). Real‐time quantitative PCR was performed using SYBR green (Yeasen, 11202ES08) on the LightCycler480 system (Roche). Gene expression levels were analyzed by ^ΔΔ^CT method with the level of 36b4 (for adipose tissues) or β‐actin (for muscles) as an internal control. Experiments were repeated three times. Sequences of primers used for real‐time quantitative PCR (qPCR) were listed in the Table S6 (Supporting Information).

### Western Blot Analysis

Proteins were extracted from tissues with RIPA buffer consisting of 50 mM Tris (pH at 7.4), 150 mM NaCl, 1% NP‐40, 0.25% sodium deoxycholate, 1 mM PMSF, 10 mM DTT and protease inhibitors. The protein concentrations were quantified using a BCA Protein Assay Kit (Beyotime Biotech, P0010). The same amount of protein was added and run on 8% sodium dodecyl sulfate polyacrylamide gel electrophoresis (SDS‐PAGE), which was later transferred to a nitrocellulose (NC) membrane (PALL, 66 485). After blocking with 5% skimmed milk, the membrane was incubated overnight at 4 °C with Atrogin‐1 (Santa Cruz, sc‐166806), MuRF‐1 (Santa Cruz, sc‐398608) or α‐tubulin (Santa Cruz, sc‐8035) antibodies. And subsequently incubated with secondary antibody at room temperature for 1 h. The images of the blots were taken using the Odyssey imaging system (LI‐COR Biotechnology).

### RNA‐Seq and Bioinformatic Analysis

RNA integrity was assessed using the RNA Nano 6000 Assay Kit of the Bioanalyzer 2100 system (Agilent Technologies, CA, USA). RNA libraries were prepared using the Fast RNA‐seq Lib Prep Kit V2 (Abclonal, RK20306) and were performed on Nova X plus sequencing instrument (Illumina). The clustering of the index‐coded samples was performed on a cBotCluster Generation System using TruSeq PE Cluster Kit v3‐cBot‐HS (Illumia) according to the manufacturer's instructions. The high‐quality reads were aligned to the mouse genome (GRCm38/mm10) using Hisat2 (v2.0.5). Feature Counts (1.5.0‐p3) was used to count the reads numbers mapped to each gene. And then FPKM of each gene was calculated based on the length of the gene and reads count mapped to this gene. Differential expression analysis was performed using the DESeq2 R package (1.20.0). *p*‐value ≤ 0.05 and Foldchange ≥ 2 were set as the thresholds for significantly differential expression. Gene Ontology (GO) enrichment analysis and Kyoto Encyclopedia of Genes and Genomes (KEGG) analysis of differentially expressed genes was implemented by the clusterProfiler R package. GO and KEGG terms with *p*‐value ≤ 0.05 were considered significantly enriched by differential expressed genes.

### Metabolomics Analysis

Adipose tissue and skeletal muscle were extracted with methanol/water/chloroform mixtures. The aqueous phase was subjected to evaporation to dryness under vacuum and the subsequent residue was reconstituted with 80% methanol for further metabolomics analysis.

Metabolomics analysis was carried out with an Acquity UPLC/Synapt XS HDMS system via positive and negative ionization. Samples were separated by a HILIC column (Waters UPLC BEH Amide 2.1 × 100 mm, i.d. 1.7 mm) using a gradient consisting of 95% acetonitrile and 5% acetonitrile with 10 mM ammonium bicarbonate and 5% ammonium hydroxide (pH 9) in both phases. Q‐TOF mass analyzer was operated at 22000 mass resolution, and the scan range covered m/z 50–1500. The untargeted metabolomics data were processed with Progenesis QI software and the ultimate output termed “feature” table with total ion ionization normalization was exported for further analysis. The databases, such as HMDB (http://www.hmdb.ca/), METLIN (https://metlin.scripps.edu/landing_page.php?pgcontent=mainPage), KEGG (https://www.kegg.jp/), as well as in‐house databases were applied for metabolite identification. Metabolite set enrichment analysis was conducted through Reactome database in Metaboanalyst (https://www.metaboanalyst.ca/).

To elucidate the metabolic crosstalk between iWAT and QU muscle in aging WT and UCP1^−/−^ mice, ^13^C_2_‐glycine (200 mg kg^−1^, sigma 283 827) or D_3_‐Creatine (100 mg kg^−1^, MedChemExpress HY‐W010388S1) was locally injected into iWAT of these mice. Subsequently, the iWAT or QU was harvested to detect labeled creatine for metabolic flux analysis via liquid chromatography‐mass spectrometry (LC/MS).

### Determination of Creatine Content and Creatine Kinase (CK) activity

The creatine content of serum and tissue were analyzed using the Creatine Assay Kit (Sigma‐Aldrich, MAK079) following the manufacturer's instruction and the colorimetric assay was measured at 570 nm using SpectraMax 190 Microplate Reader (Molecular Devices). CK activity in the serum samples was assayed using a Creatine Kinase test kit (A032‐1‐1, Nanjing Jiancheng) following the manufacturer's instruction.

### LDH Isoenzyme Analysis

Skeletal muscles were homogenized in a solution of 0.9% NaCl, 5 mm Tris‐HCl (pH7.4), and the lysates were centrifuged for 0.5 h at 12000 g to remove the cellular debris. One‐hundred micrograms of protein was loaded onto a 6% nondenaturing polyacrylamide gel (native PAGE) (6%(vol/vol) acrylamide, 37.5:1)^[^ ^73^ ^]^ and performed LDH isoenzyme analysis as previously reported.^[^ ^74^, ^75^ ^]^ Following electrophoresis, the gel was placed in 10 ml of staining solution containing 0.1 M sodium lactate, 0.1 M Tris‐HCl (pH8.6), 1.5 mM NAD, 10 mM NaCl, 5 mM MgCl_2_, 0.03 mg ml^−1^ phenazinmethosulphate, and 0.25 mg ml^−1^ nitro blue tetrazolium. The staining was observed after 30 min. Protein extracted from mouse heart (hrt) was used as a positive control.

### Immunofluorescence

Skeletal muscles were freshly isolated from mice, flash frozen in OCT and cold isopentane, and cut at 10 µm per section. Muscle fibers were stained with antibody against MHCI (BA‐D5, Developmental Studies Hybridoma Bank). Briefly, frozen sections were fixed in ice‐cold 4% PFA for 15 min and 0.5% Triton X‐100 for 10 min to permeabilize cell membrane. The samples were blocked with PBS containing 10% normal goat serum for 1 h at room temperature and further incubated with MHCI antibody overnight at 4 °C. The following day, the samples were incubated with goat anti‐mouse Fluorescein Isothiocyanate secondary antibody at room temperature for 1 h. After washing, the staining images were captured with confocal microscope.

C2C12 myotubes were fixed with 4% paraformaldehyde (PFA) and washed with PBS three times before MyHC staining. C2C12 myotubes were permeabilized with PBS containing 0.2% Triton X‐100 for 15 min and then washed three times with PBS, and then blocked with PBS containing 10% normal goat serum for 1 h at room temperature and further incubated with mouse anti‐myosin (MyHC, MF‐20, DSHB) overnight at 4 °C. The following day, the cells were incubated with ABflo 488‐conjugated goat anti‐mouse IgG (H+L) (AS076, ABclonal) at room temperature for 1 h. After washing, the staining images were captured with fluorescence microscope.

### Histological Analysis

Frozen serial transverse cryosections (10 µm) from the QU, GAS and TA were cut with a cryostat and mounted on glass slides. Slides were fixed with pre‐cooled 4% PFA overnight at 4 °C and then washed three times with PBS. The slides were stained with H&E. Slides were analyzed using a microscope (Olympus) at the indicated magnification and images were captured by a digital camera (Olympus). For the cross‐sectional areas (CSA) quantification of muscle fiber, 5 random fields in each slide and measured at least 300 fibers using Image‐Pro Plus 6.0 software was selected.^[^ ^76^, ^77^ ^]^

### Statistical Analysis

Data were analyzed with GraphPad prism Software 8 and shown as means ± SEM. Student's t test or ANCOVA^[^ ^71^ ^]^ analysis were used for comparison between two groups. *p* < 0.05 was considered as significant difference. The P‐values were designated as *p < 0.05 and **p < 0.01.

## Conflict of Interest

The authors declare no conflict of interest.

## Author Contributions

J.Q., Y.G., and X.G. contributed equally to this work. X.M., L.X. and C.X. performed conceptualization; X.M., L.X., J.Q. and D.W performed methodology; J.Q., Y.G., X.G., Z.L., W.Z., Z.L., J.Z., Y.C., J.L., S.Y., S.X., J.C., D.W., J.Y., M.G., W.Z., J.Z., S.W., and Y.W. performed investigation; C.X., L.X., and X.M. provided resources; X.M., L.X. and J.Q. wrote and edited; X.M., L.X., C.H. and C.X performed project administration; X.M., L.X. and C.X. performed funding acquisition; X.M., L.X. and C.X supervised. All authors have read and approved the manuscript.

## Supporting information



Supporting Information

Supplemental Tables 1‐6

## Data Availability

The data that support the findings of this study are available from the corresponding author upon reasonable request.
