# Supplementary material for: Ucp1 Ablation Improves Skeletal Muscle Glycolytic Function in Aging Mice
Source: Adv Sci (Weinh). 2024 Nov 21;12(2):2411015. doi: 10.1002/advs.202411015 (PMC11727132; doi:10.1002/advs.202411015)
Supplement: Supplementary file 1 — Supporting Information [file ADVS-12-2411015-s001.docx]

**Supporting Information**

**Figure S1. Liver phenotype and serum parameters of WT and UCP1 deficient mice.**

(A, B) Gene expression of UCP1 in iWAT (A) and MuRF-1 in GAS (B) of different ages in mice.

(C) H&E staining and relative adipocyte sizes of eWAT from aging UCP1^+/+^ and UCP1^-/-^ mice. Scar bar is 45 μm.

(D-H) Liver weights (D), hepatic TG levels (E), H&E staining of liver (F), serum lipid parameters (G, H) from aging UCP1^+/+^ and UCP1^-/-^ mice.

(I, J) Locomotor activity (I) and food intake (J) were measured using CLAMS in aging UCP1^+/+^ and UCP1^-/-^ mice. Data are presented as mean ± SEM and *P <0.05, **P <0.01 compared to aging UCP1^+/+^ group. n=8 per group.

**Figure S2. Histological Analysis of GAS and TA muscles from aging UCP1^-/-^ mice compared to UCP1^+/+^ mice.**

(A, B) Representative images of H&E staining and relative fiber sizes of GAS (A) and TA (B) from aging UCP1^+/+^ and UCP1^-/-^ mice. Scale bar is 45 µm. n=8 per group.

**Figure S3. UCP1 deficiency improves GAS and TA muscle function in aging mice.**

(A-D) Gene and protein expression analysis of muscle atrophy markers of GAS (A, B) and TA (C, D) from aging UCP1^+/+^ and UCP1^-/-^ mice.

(E, F) Gene expression analysis of glucose uptake and glycolysis of GAS (E) and TA (F) from aging UCP1^+/+^ and UCP1^-/-^ mice.

(G) Gene expression analysis of mitochondrial function of QU from aging UCP1^+/+^ and UCP1^-/-^ mice.

(H, I) Polyacrylamide gel electrophoresis analysis of LDH isoenzyme activity of GAS (H) and TA (I) from aging UCP1^+/+^ and UCP1^-/-^ mice.

(J) Relative creatine kinase activity of serum from aging UCP1^+/+^ and UCP1^-/-^ mice.

Data are presented as mean ± SEM and *P <0.05, **P <0.01 compared to aging UCP1^+/+^ group. Hrt: heart as positive control. n=8 per group.

**Figure S4. Phenotype of young WT and UCP1 deficient mice.**

(A-C) Body weight (A), lean mass (B) and fat mass (C) of young UCP1^+/+^ and UCP1^-/-^ mice (3-month-old).

(D) Grip strength analysis of young UCP1^+/+^ and UCP1^-/-^ mice.

(E) Weight of QU, GAS and TA from young UCP1^+/+^ and UCP1^-/-^ mice.

(F-H) Representative images of H&E staining and relative fiber sizes of QU (F), GAS (G) and TA (H) from young UCP1^+/+^ and UCP1^-/-^ mice. Scale bar is 45 µm.

Data are presented as mean ± SEM and *P <0.05, **P <0.01 compared to UCP1^+/+^ group. n=5 per group.

**Figure S5. Cardiac function analysis in aging WT and UCP1 deficient mice.**

(A) Representative echocardiograms from aging UCP1^+/+^ and UCP1^-/-^ mice.

(B-G) Measurements of heart functionality including hemodynamics (B), ejection fraction (C), fractional shortening (D), left ventricular internal diameter in diastole (LVIDD) (E) and systole (LVIDS) (F) and stroke volume (G) from aging UCP1^+/+^ and UCP1^-/-^ mice.

(H) Gene expression analysis of cardiac hypertrophy and fibrosis marker in heart from aging UCP1^+/+^ and UCP1^-/-^ mice.

Data are presented as mean ± SEM and *P <0.05, **P <0.01 compared to control group. LVIDS: left ventricular internal diameter in systole; LVIDD: left ventricular internal diameter in diastole. n=4 per group.

**Figure S6. Brown adipocytes did not crosstalk with myotubes to attenuate atrophy.**

(A) Schematic illustration of co-culture system of brown adipocytes and myotubes.

(B-E) Gene expression analysis of glucose metabolism (B), representative images of MyHC staining (C), mRNA (D) and protein levels (E) of muscle atrophic markers of dexamethasone (DEX)-treated C2C12 myotubes cultured with or without conditioned medium (CM) from differentiated primary brown adipocytes of aging UCP1^+/+^ and UCP1^-/-^ mice.

n =3 biological replicates for each group; Data are presented as mean ± SEM and *P <0.05, **P <0.01 compared to control group. CM: Conditioned medium.

**Figure S7. Creatine metabolism is altered in GAS and TA muscles of aging UCP1 deficient mice.**

(A, B) Enrichment analysis of down-regulated metabolites in QU (A) and iWAT (B) from aging UCP1^-/-^ mice compared to UCP1^+/+^ mice.

(C) Overlapped down-regulated metabolites between QU and iWAT.

(D, E) Creatine levels in GAS (D) and TA (E) from aging UCP1^+/+^ and UCP1^-/-^ mice.

(F, G) Gene expression analysis of creatine synthesis and transport in GAS (F) and TA (G) from aging UCP1^+/+^ and UCP1^-/-^ mice.

(H, I) Gene expression analysis of creatine metabolism and creatine levels in liver from aging UCP1^+/+^ and UCP1^-/-^ mice.

(J, K) Gene expression analysis of Slc6a8 in skeletal muscle from young and aging mice.

Data are presented as mean ± SEM and *P <0.05, **P <0.01 compared to control group. n=4 or 8 per group.

**Figure S8. β-guanidinopropionic acid (β-GPA) treatment deteriorates GAS and TA skeletal muscle function in aging UCP1^-/-^ mice.**

(A, B) Creatine levels in GAS (A) and TA (B) from aging UCP1^-/-^ mice treated with β-GPA or vehicle.

(C, D) Representative images of H&E staining and relative fiber sizes of GAS (C) and TA (D) from aging UCP1^-/-^ mice treated with β-GPA or vehicle. Scale bar is 45 µm.

(E-G) Gene expression analysis of mitochondrial function of QU (E) and glucose metabolism in GAS (F) and TA (G) from aging UCP1^-/-^ mice treated with β-GPA or vehicle.

(H, I) WB analysis of muscle atrophic markers in GAS (H) and TA (I) from aging UCP1^-/-^ mice treated with β-GPA or vehicle.

(J-L) Changes in body weight (J), lean mass (K) and fat mass (L) of aging UCP1^-/-^ mice injected with control or β-GPA.

(M) Weight of adipose tissues of aging UCP1^-/-^ mice injected with control or β-GPA.

Data are presented as mean ± SEM and *P <0.05, **P <0.01 compared to control group. β-GPA: β guanidinopropionic acid. n=8 per group.

**Figure S9. UCP1 inhibitor α-CD administration leads to improved GAS and TA muscle functionality in aging mice.**

(A, B) Creatine levels in GAS (A) and TA (B) from aging mice treated with α-CD or vehicle.

(C, D) Gene expression analysis of creatine synthesis and transport in GAS (C) and TA (D) from aging mice treated with α-CD or vehicle.

(E) Gene expression analysis of mitochondrial genes of iWAT from aging mice treated with α-CD or vehicle.

(F, G) Representative images of H&E staining and relative fiber sizes of GAS (E) and TA (F) from aging mice treated with α-CD or vehicle. Scale bar is 45 µm.

(H-J) Gene expression analysis of mitochondrial function of QU and glucose metabolism in GAS (H) and TA (I) from aging mice treated with α-CD or vehicle.

(K-N) qPCR and WB analysis of muscle atrophy markers in GAS (J, K) and TA (L,M) from aging mice treated with α-CD or vehicle.

Data are presented as mean ± SEM and *P <0.05, **P <0.01 compared to control group. α-CD: α-cyclodextrin. n=8 per group.

**Figure S10. UCP1 inhibitor α-CD administration did not influence body weights and fat weights of aging mice.**

(A, B) Oxygen consumption (VO_2_, A), carbon dioxide excretion (VCO_2_, B), locomotor activity (C) and food intake (D) in CLAMS of aging mice treated with α-CD or vehicle.

(E-G) Changes in body weight (E), lean mass (F) and fat mass (G) of aging mice treated with α-CD or vehicle.

(H) Weights of adipose tissues from aging mice treated with α-CD or vehicle.

(I) Gene expression analysis of mitochondrial function of BAT from aging mice treated with α-CD or vehicle.

(J-L) Liver weights (J), hepatic TG levels (K) and H&E staining of liver (L) from aging mice treated with α-CD or vehicle. Scar bar is 45 μm.

Data are presented as mean ± SEM and *P <0.05, **P <0.01 compared to aging control group. α-CD: α-cyclodextrin. n =8 per group.

**
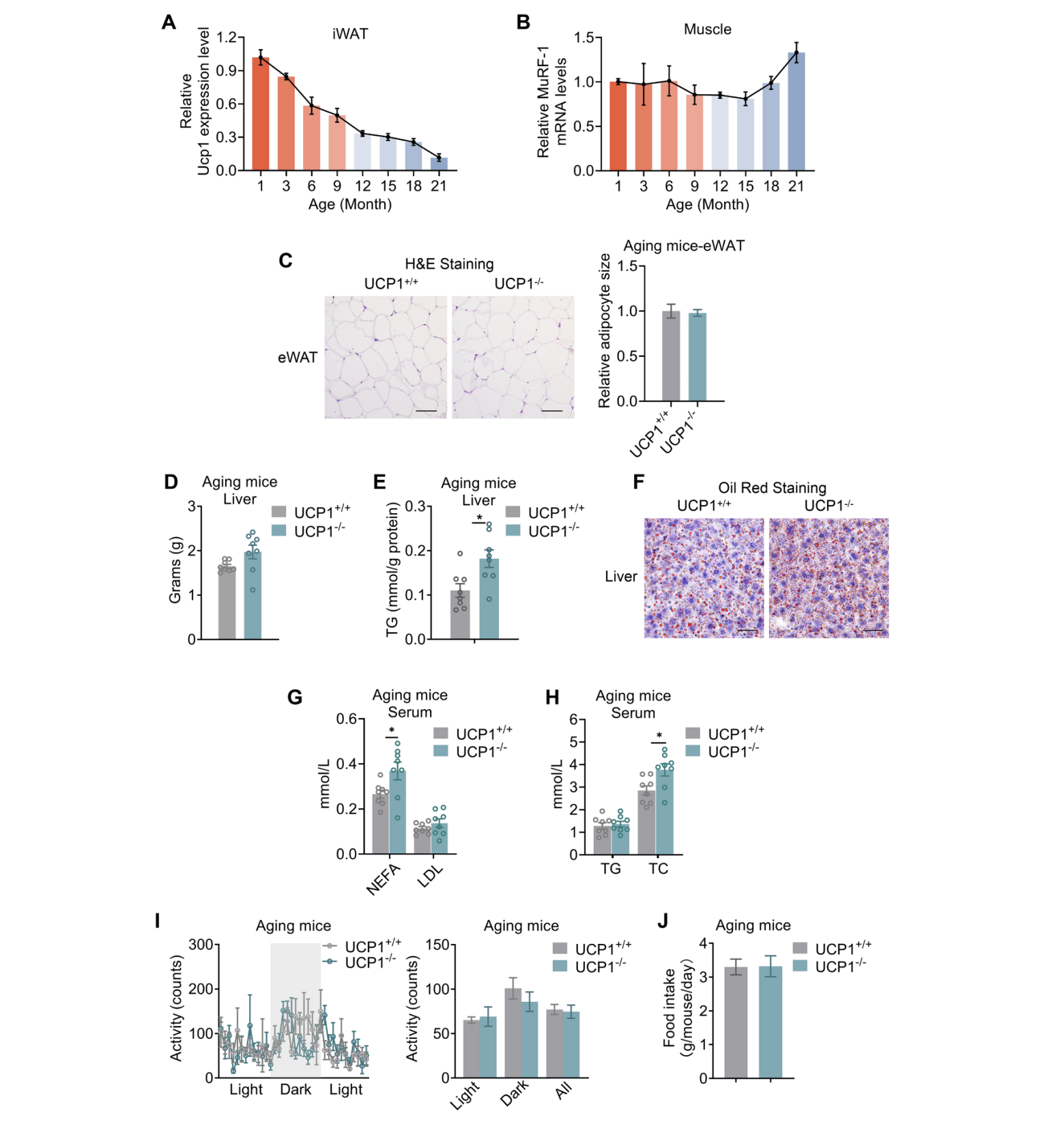
**

**Figure S1**

**
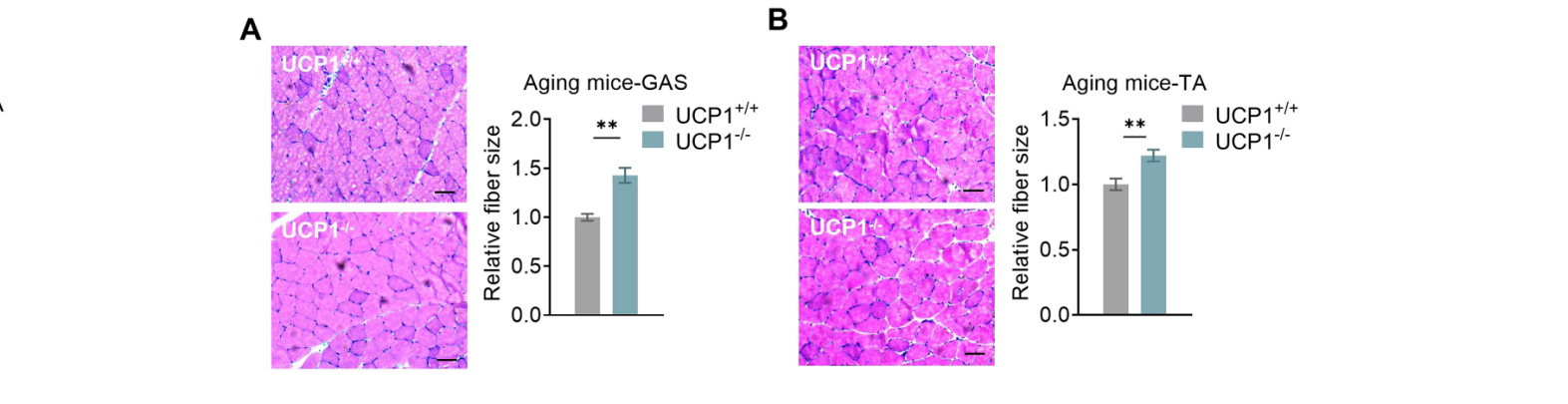
**

**Figure S2**

**
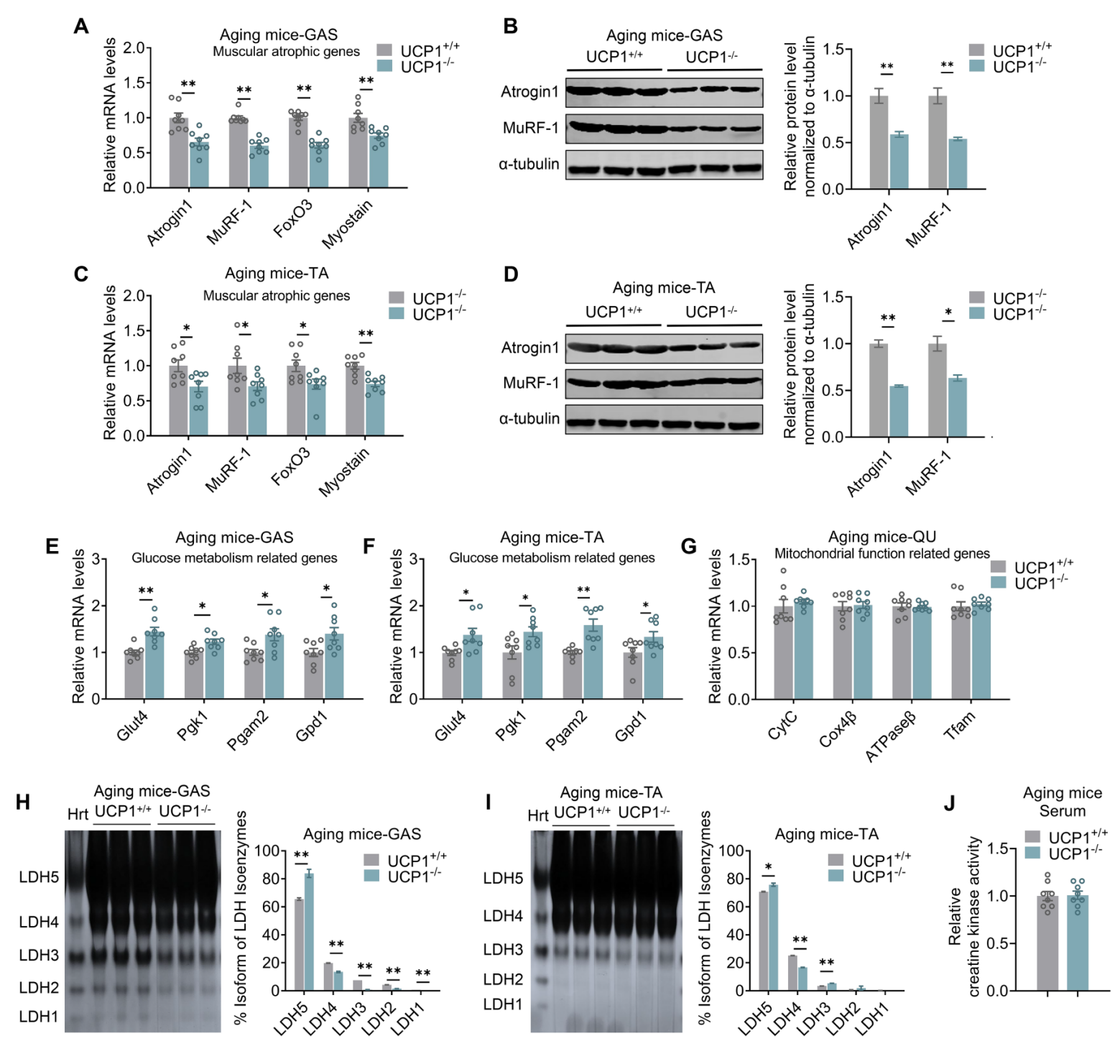
**

**Figure S3**

**
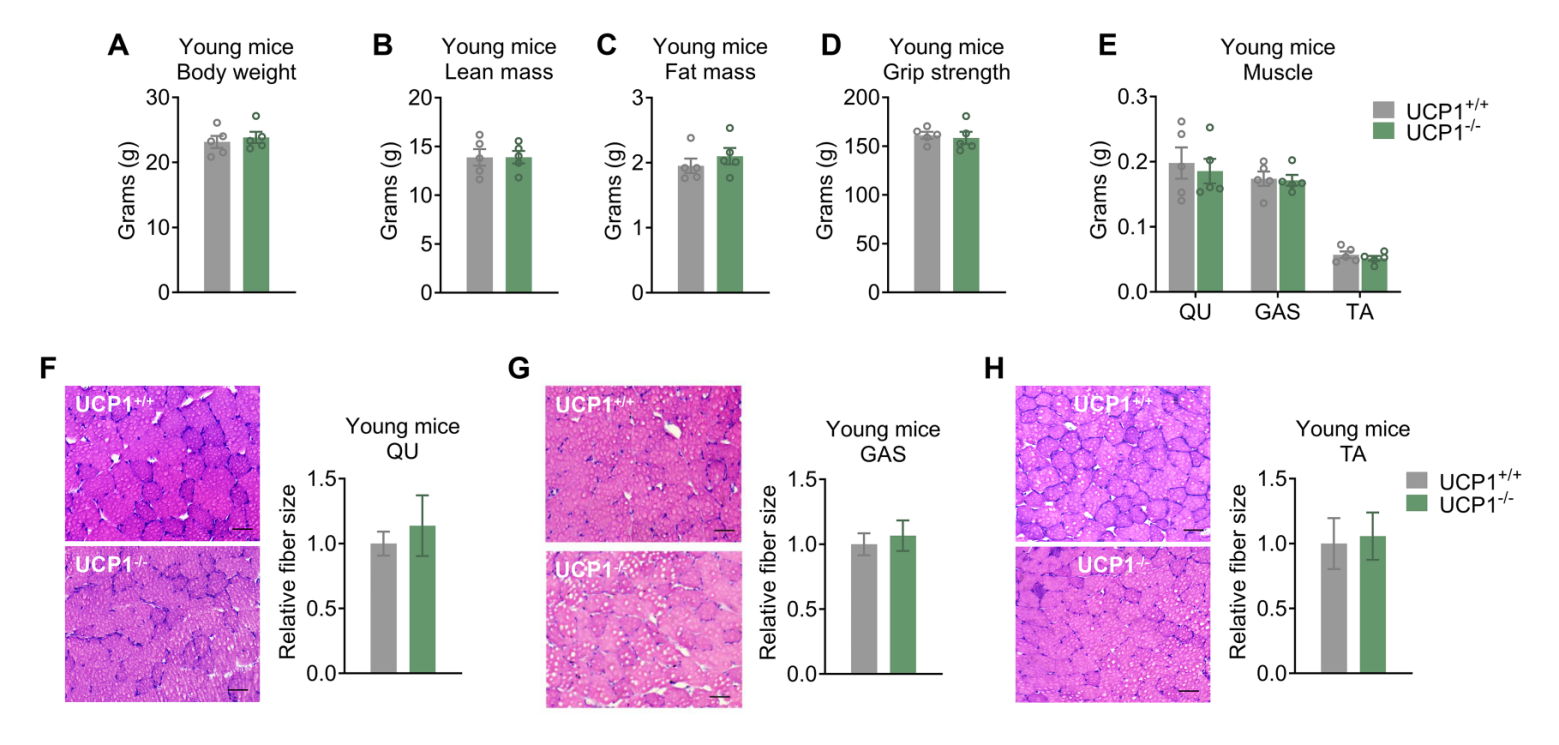
**

**Figure S4**

**
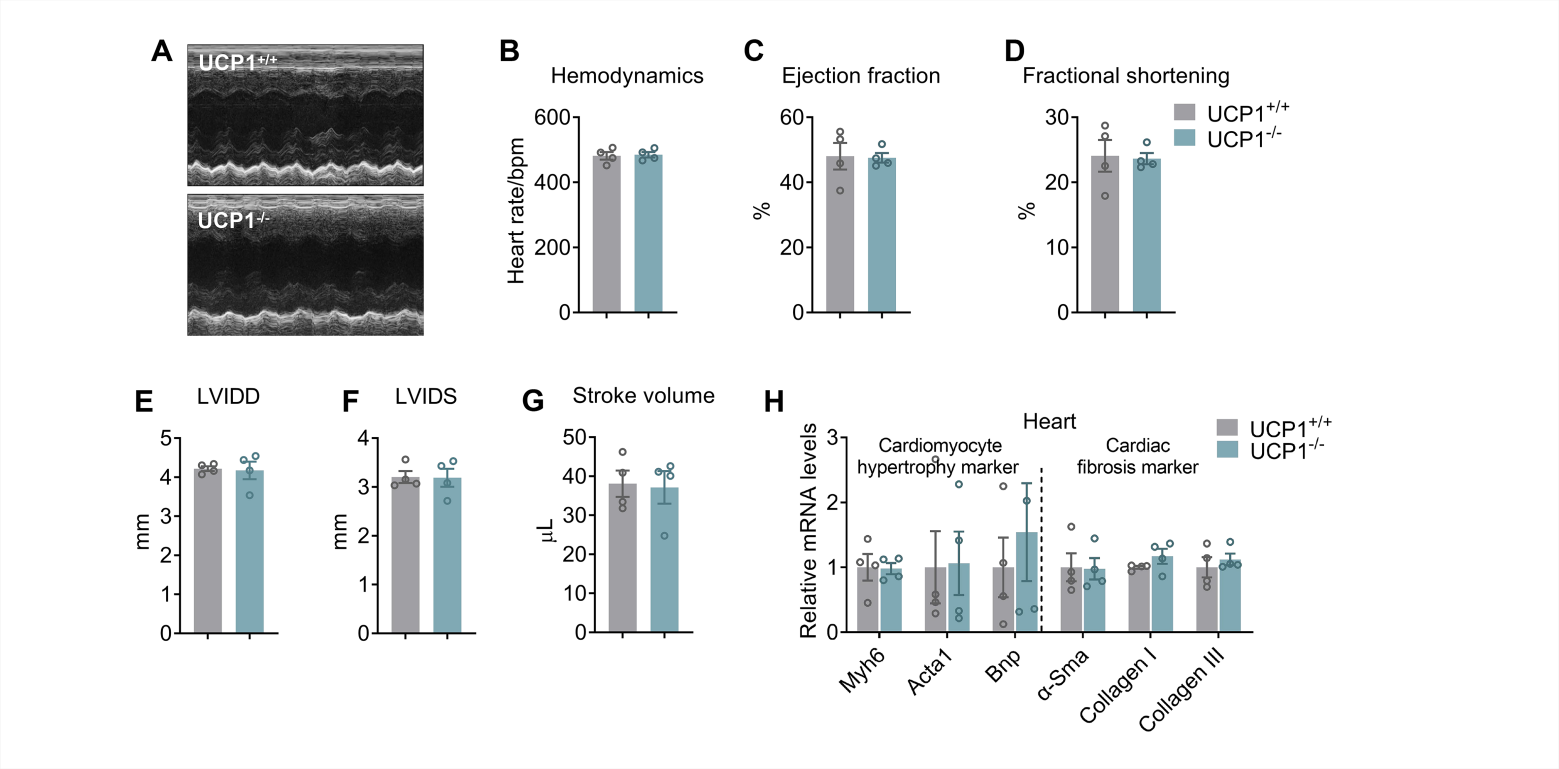
**

**Figure S5**

**
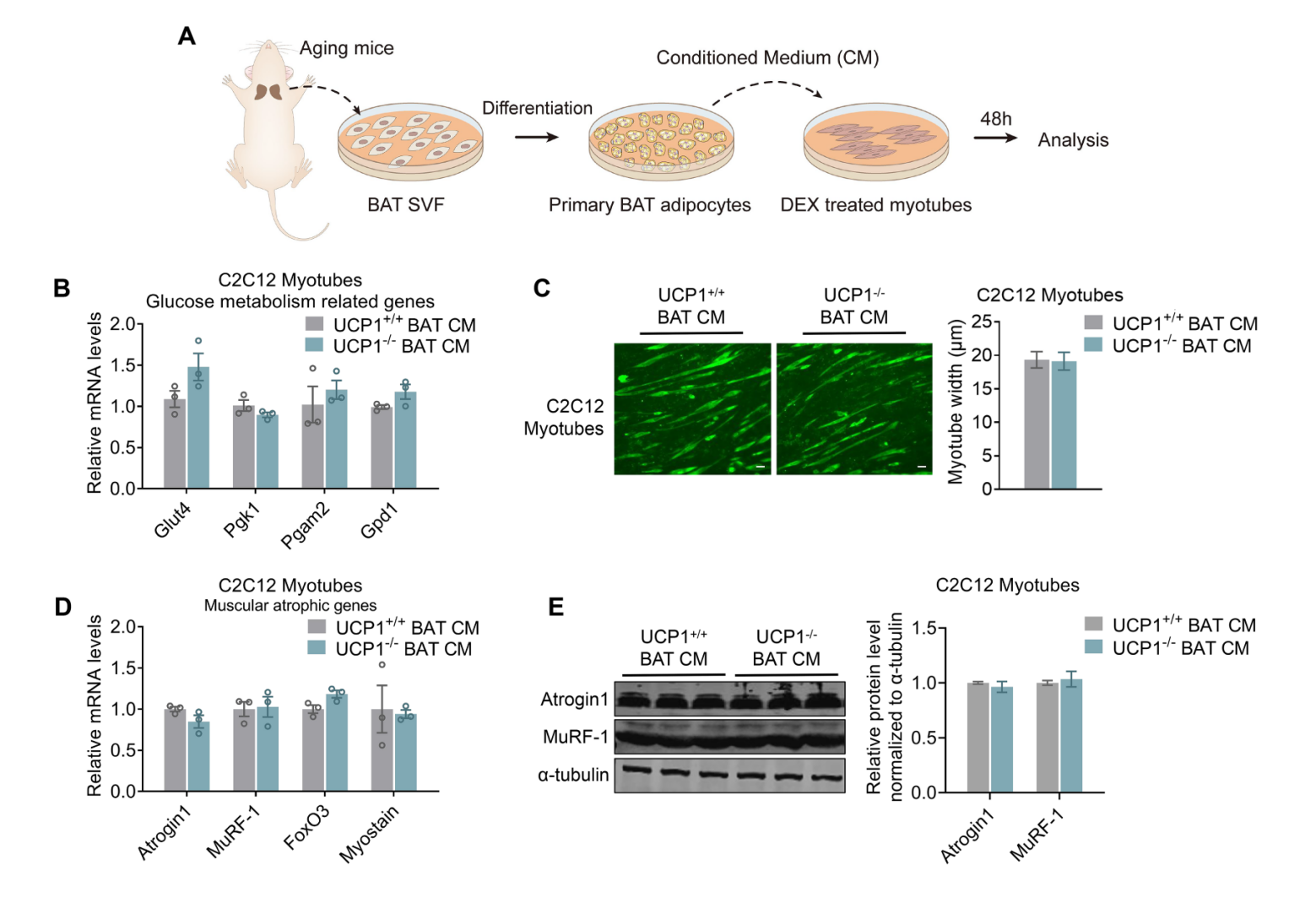
**

**Figure S6**

**
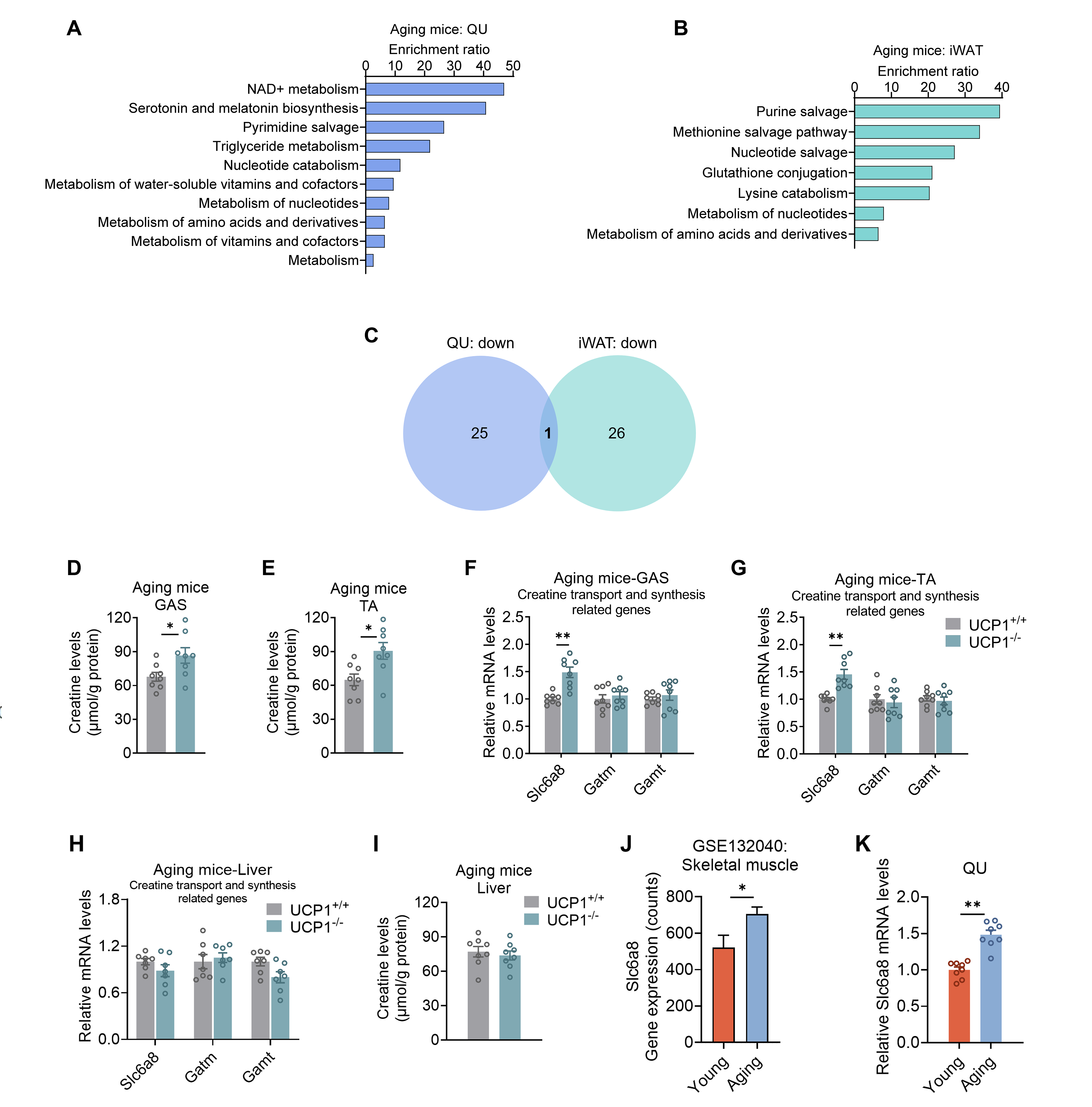
**

**Figure S7**

**
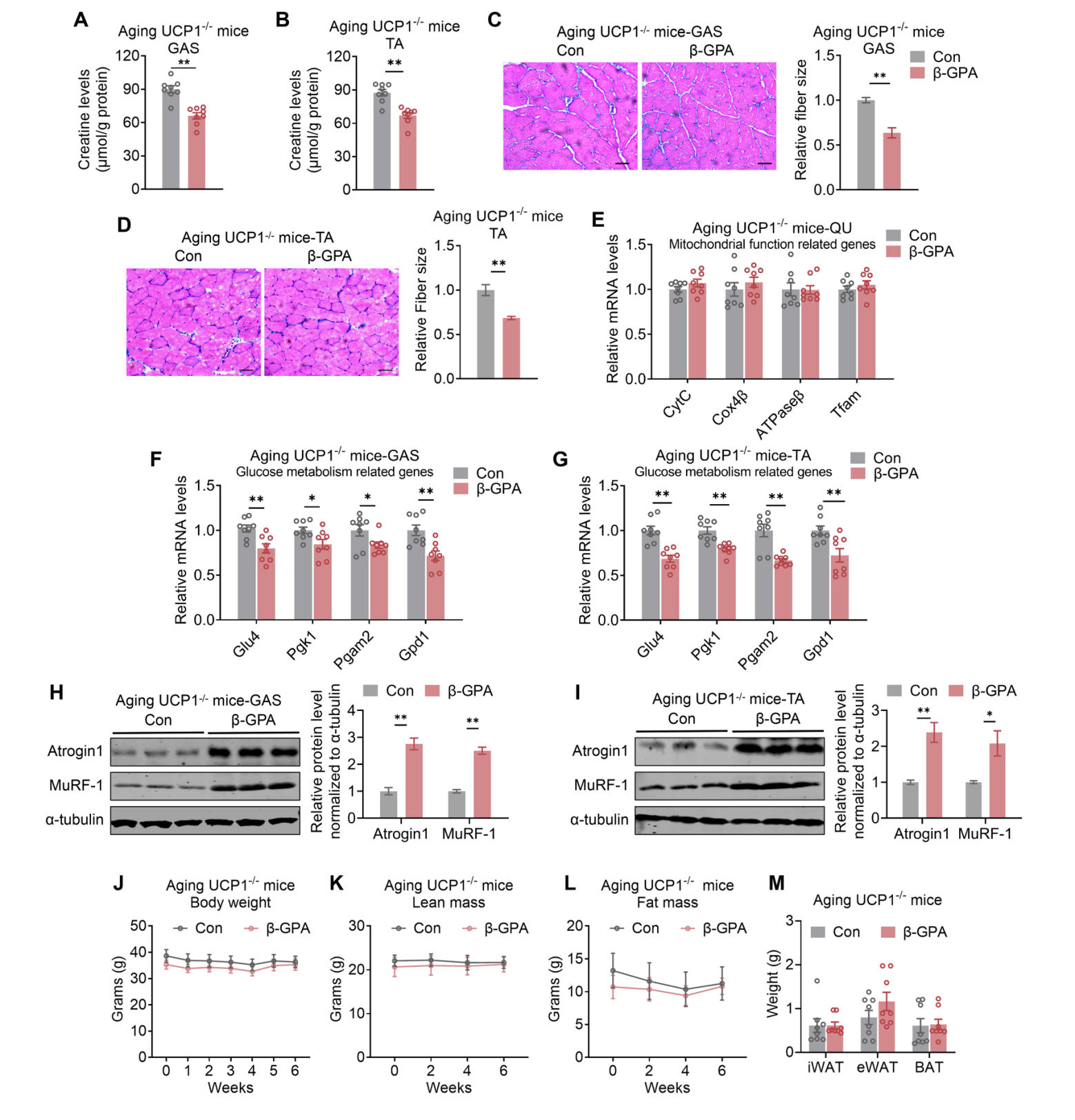
**

**Figure S8**

**
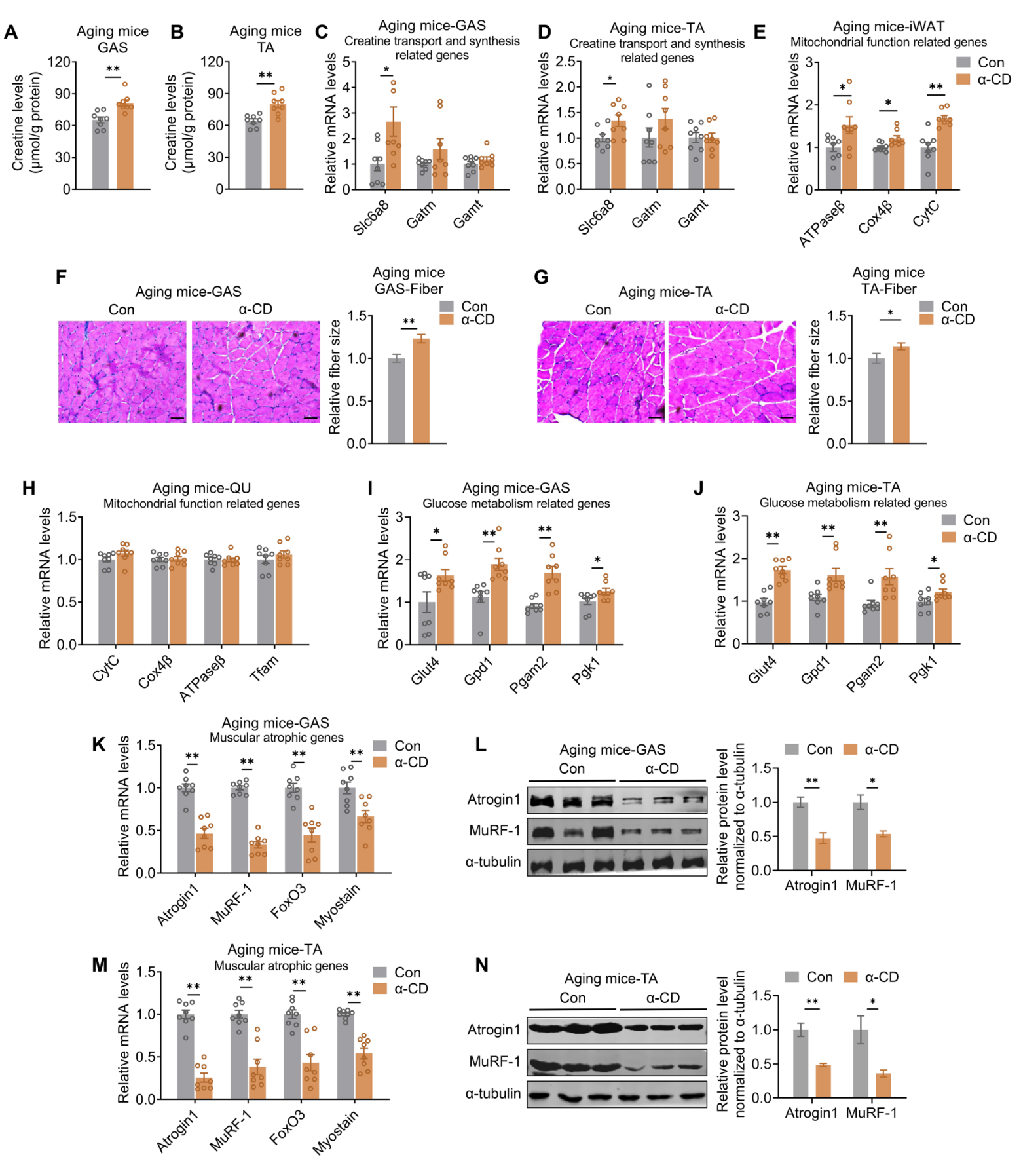
**

**Figure S9**

**
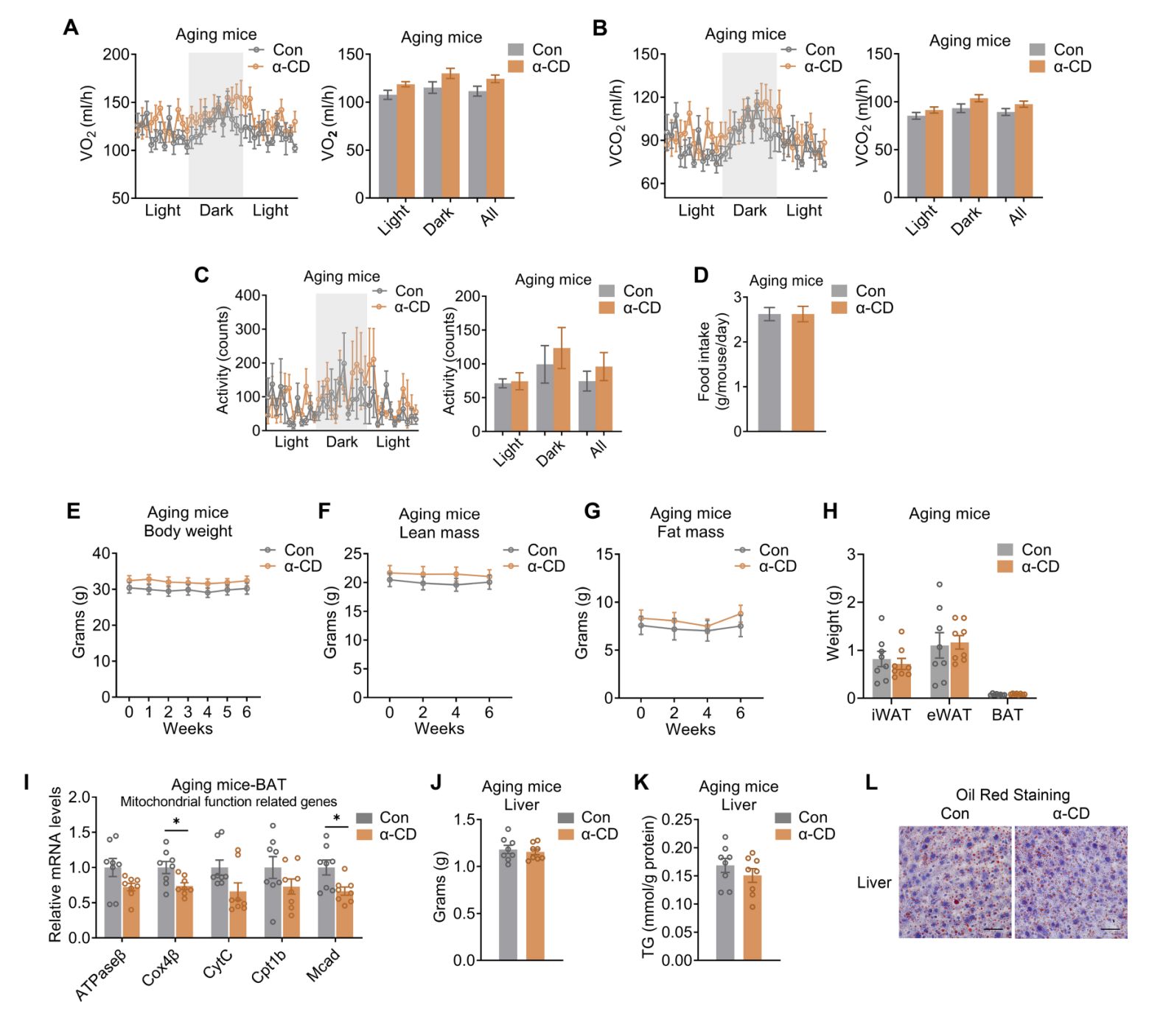
**

**Figure S10**
